# Coronary Drug-Coated Balloons as Brief-Contact Drug-Delivery Platforms: Engineering, Tissue Pharmacokinetics, and Angiographic/Imaging Response

**DOI:** 10.3390/medicina62101932

**Published:** 2026-10-07

**Authors:** Grigorios Tsigkas, Antonios Rigas Papapanagiotou, Aggelos Papanikolaou, Athanasios Papageorgiou, Spyridon Graidis, Georgios Vasilagkos, Anastasia Mavromati, Alexandros Dedes, Panagiota Kravvariti, Athanasios Moulias, Michail Papafaklis, Antonios Karanasos, Periklis Davlouros

**Affiliations:** 1 Cardiology Department, General University Hospital of Patras, 26504 Rio, Greece; gregtsig@upatras.gr (G.T.); thanasispapag95@gmail.com (A.P.); spyrosgraid@gmail.com (S.G.); george.vasilagos@hotmail.com (G.V.); natmavr@yahoo.gr (A.M.); alexandros.a.dedes@gmail.com (A.D.); penykravvariti@gmail.com (P.K.); dramoulias@live.com (A.M.); m.papafaklis@yahoo.com (M.P.); akaranasos@hotmail.com (A.K.); pdav@upatras.gr (P.D.); 2 Cardiology Department, Hippokration General Hospital, 114 Vasilissis Sofias Avenue, 11527 Athens, Greece; agepap25@otenet.gr

**Keywords:** drug-coated balloon, coronary artery disease, paclitaxel, sirolimus, drug delivery, tissue pharmacokinetics, coating technology, intravascular imaging, late lumen loss, late lumen enlargement

## Abstract

*Background and Objectives*: Coronary drug-coated balloons (DCBs) deliver antiproliferative therapy during a single inflation without leaving a permanent scaffold or implanted drug reservoir. Their performance therefore depends on preservation of the coating during delivery, efficient vessel-wall transfer, and sufficient tissue exposure after balloon removal. This review critically examines the evidence for platform engineering, tissue exposure, and angiographic or intravascular imaging response. *Materials and Methods*: This narrative review synthesized English-language experimental, pharmacokinetic, imaging, and clinical evidence identified through the original PubMed, Scopus and Google Scholar searches and a documented PubMed search during revision on 9 September 2026. Exact queries, record dispositions and descriptive study appraisals are supplied; conference abstracts, preprints and unpublished data were excluded. *Results*: DCB performance is platform-specific rather than determined by drug class alone. Within both paclitaxel- and limus-based devices, excipient chemistry, coating or carrier architecture, release behavior, and retention strategy may influence drug delivery. Bench and computational studies identify potential transfer and drug-loss pathways, pharmacokinetic studies measure tissue exposure, and imaging and clinical studies assess morphology and outcomes separately. The cited human studies do not quantify coronary tissue drug exposure. Findings from unrelated platforms and models cannot establish a causal delivery-to-outcome pathway. *Conclusions*: Coronary DCBs should be evaluated as integrated, device-specific drug-delivery platforms. Standardized studies linking engineering performance and tissue exposure with imaging and clinical outcomes are needed.

## 1. Introduction

Drug-coated balloon (DCB) angioplasty differs from drug-eluting stent (DES) implantation because treatment is delivered without a persistent scaffold or polymer reservoir. Whereas DES release drug over days to months, DCBs initiate drug transfer during brief inflation, with tissue distribution and retention continuing after balloon removal [1]. This narrow window makes the initial device–vessel interaction decisive: coating stability, release kinetics, wall contact, acute transfer, and post-transfer retention all interact with drug pharmacology [1,2,3].

The biological effect of DCBs is determined by drug physicochemistry, excipient microstructure, coating resilience during tracking, balloon expansion and apposition, tissue uptake, and washout. Experimental studies examine these delivery processes [4,5,6,7,8], but their clinical consequences require direct investigation in the relevant device and lesion setting. Accordingly, nominal drug load and active drug class are incomplete descriptors of performance; the same drug may behave differently when paired with different excipients, coating architectures, carrier systems, or release mechanisms.

### Objective of This Review

Marchetta et al. have already reviewed DCB engineering, pharmacology, and the mechanisms proposed to explain angiographic outcomes [9]. The present review shares that subject matter. Its narrower purpose is to critically examine what has actually been measured for individual coronary DCB platforms, distinguish preclinical tissue pharmacokinetics from human imaging and clinical endpoints, and identify the untested links that prevent attribution of clinical outcomes to particular delivery characteristics. Tables 1–3 and S1 organize these distinctions by platform, study design, and lesion setting; they do not constitute a causal model or a comparative ranking of devices. The DCB-ARC documents provide the separate context of clinical indications and standardized endpoints [1,10].

## 2. Scope and Synthesis Approach

This narrative review synthesizes experimental, pharmacokinetic, imaging and clinical evidence on coronary DCB delivery and vascular response. PubMed and Scopus were searched through 1 May 2026, supplemented by Google Scholar searches and a targeted update on 30 June 2026. Terms covered drug-coated or drug-eluting balloons, paclitaxel, sirolimus/limus, coatings, carriers, transfer, tissue uptake or retention, pharmacokinetics, particle loss, OCT, IVUS, late lumen loss or enlargement, ISR and coronary intervention.

During revision, two additional PubMed searches were conducted via NCBI E-utilities on 9 September 2026, covering publications through that date. Exact final search strings, native retrieval responses, deduplication, screening reasons and source assessments are provided in Supplementary Methods S1. Conference abstracts, preprints, unpublished material and non-English publications were excluded. For the original narrative synthesis, studies were selected through collaborative discussion and consensus, prioritizing reports identifying a device or formulation and reporting engineering data, quantitative drug transfer or retention, or angiographic, intravascular imaging or clinical endpoints. Supplementary Table S1 provides study-level critical appraisal of cited reports, recording design, device or formulation, experimental or clinical setting, comparator, measurements and inference-specific limitations.

Direct tissue drug measurements are distinguished from computational estimates, histological findings, OCT appearances, angiographic endpoints and clinical events. Peripheral experiments are interpreted within their original models and do not establish coronary drug transfer or clinical applicability. Findings from different devices, models or populations are considered separately, without combining them to establish a causal pathway.

## 3. Mechanistic Foundations of DCB Therapy

### 3.1. Drug Physicochemistry as a Design Constraint

Drug physicochemistry is central to DCB performance, but it functions as a design constraint rather than as the sole determinant of efficacy. Unlike DES, DCBs must transfer, distribute, and retain drug after brief balloon-artery contact. Lipophilicity, membrane partitioning, intracellular target engagement, tissue binding, particulate or carrier behavior, and washout kinetics influence how much delivery support each platform requires [1,5,11,12,13,14].

Paclitaxel has physicochemical and intracellular properties that may support brief-contact transfer, including high lipophilicity, tissue partitioning, and microtubule binding [11,12]. However, the magnitude, distribution, and persistence of tissue exposure remain dependent on the coating architecture and excipient behavior of the individual platform, as well as on lesion preparation, wall contact, and washout. Paclitaxel should therefore not be regarded as intrinsically effective independent of platform design.

Limus drugs are potent antiproliferative agents, but contemporary coronary DCB platforms generally use specific delivery architectures to support tissue entry and post-contact exposure. These include carrier-mediated uptake, crystalline deposition, phospholipid encapsulation, porous infusion, biodegradable micro-reservoirs, and related persistence-oriented strategies [13,14,15,16,17,18,19,20]. Observed differences among sirolimus and other limus balloons should therefore be interpreted in relation to the individual delivery architecture and design and results of the available studies, rather than as a uniform limus-class effect.

Direct preclinical comparisons should be interpreted at the platform and model level. A porcine coronary study compared MagicTouch sirolimus-coated balloon (SCB), SELUTION SLR SCB, AGENT paclitaxel-coated balloon (PCB), and plain old balloon angioplasty (POBA). All DCBs were associated with minimal neointimal formation in that model, but distal findings differed. Embolic material was reported in 15%, 25%, 36%, and 6% of histological sections, respectively, while downstream myocyte necrosis/scarring was observed in 21% of paclitaxel-balloon sections and in none of the other groups [14]. These are hypothesis-generating porcine safety and distribution signals; they should not be extrapolated directly to clinical harm.

A platform-level evaluation should distinguish antiproliferative efficacy from vascular-healing and downstream-safety considerations. In experimental studies, paclitaxel’s short-contact pharmacology coexists with potential concerns related to particulate transfer, off-target loss, downstream exposure, local cytotoxicity, and delayed healing [11,14]. Limus-based platforms combine cytostatic pharmacology with engineered persistence; however, any vascular-healing advantage remains dependent on the individual platform, achieved exposure, and experimental model and should not be assumed to represent a class-wide clinical effect [15,16,17,18,19]. Local efficacy and vascular response depend on the integrated behavior of the drug, excipient or carrier, coating architecture, tissue transfer, retention, and washout [4,7,8,21,22,23].

### 3.2. Tissue Pharmacokinetics and Effective Dose

In balloon-based delivery, nominal drug load is not equivalent to biologically active mural exposure. Loaded dose, coating retained after delivery, drug released at inflation, drug transferred into the vessel wall, drug retained over time, and effective exposure are distinct quantities. Coating may be lost during handling, guide-catheter and guide extension passage, tortuous tracking, lesion crossing, balloon expansion, deflation, withdrawal, or early washout [4,7,8,21].

Clinically relevant exposure is best conceptualized as the fraction of drug that reaches and persists in biologically relevant mural compartments long enough to influence restenotic biology. Because inhibitory thresholds are not routinely defined for each named coronary platform, nominal surface dose should not be used as a surrogate for effective local exposure.

Bench and translational studies illustrate pre-lesion and off-target variability under model-specific conditions; they do not establish clinical efficacy or harm. In an artificial robotic abrasion test of eight peripheral paclitaxel DCB products, visible coating loss ranged from 2.25% to 45.73% in the deflated state and from 1.66% to 40.41% after inflation [21]. These data indicate large between-device differences in mechanical vulnerability, but they are not direct coronary tissue-transfer or pharmacokinetic measurements.

Tscheuschner et al. studied the peripheral PearlFlow paclitaxel/BTHC balloon in porcine superficial femoral arteries, with 5 mm × 60 mm devices and 3 min inflations, alongside separate bench and computational experiments [24]. The experiments examined off-target loss during peripheral delivery. Device dimensions, vascular anatomy, access conditions, and inflation duration differ from coronary use. Consequently, the reported loss fractions and tissue concentrations are not used here to estimate coronary delivery, exposure, or safety.

Effective dose should therefore be treated as a post-handling, post-tracking, post-release, and post-transfer variable. The relevant question is how much drug remains available at the treated segment, enters the vessel wall, and persists in biologically active form.

### 3.3. Transmural Distribution and Lesion Dependence

Drug distribution within the arterial wall is heterogeneous and modified by lesion substrate. Atherosclerotic tissue can alter uptake, wall adhesion, intramural diffusion, binding, and washout in an excipient- and platform-dependent manner [22,23]. Computational models can identify mechanisms and generate hypotheses, while human imaging and clinical studies characterize morphology and outcomes without measuring drug distribution.

In an excipient-plaque model, a urea-based formulation simulated higher tracking loss than a butyryl-trihexyl citrate comparator (35.5% vs. 8.13%) but higher modeled arterial uptake, while calcified and non-calcified atheroma altered transmural propagation and retentive potential [23]. These are simulation outputs dependent on the modeled formulation, geometry, and transport parameters; they are not measured coronary uptake or evidence of clinical benefit.

Clinical and imaging data suggest that lesion substrate is relevant; the reported observational findings are associative. In an optical coherence tomography/optical frequency-domain imaging (OCT/OFDI) analysis of 328 de novo DCB-treated lesions, target-lesion failure occurred in 9.5% and was independently associated with hemodialysis, greater maximum calcium arc per 90-degree increment, and absence of post-percutaneous coronary intervention (PCI) medial dissection [25]. These findings link calcific burden and procedural morphology to DCB outcomes, but no tissue drug concentration was measured. Within the same imaging framework, lower event rates were observed when maximum calcium arc was <180°, although calcium thickness, longitudinal calcium length, circumferential distribution, and the feasibility of plaque modification should also be considered when assessing lesion preparation; their effects on drug access were not measured in this cohort [25].

## 4. Excipient Chemistry and Adhesion Switching

Excipients are functional delivery components, not passive additives. Without a permanent polymer reservoir, they must help preserve coating during delivery, permit rapid or triggered release during inflation, and support local drug availability after deflation [4,7,8,26,27]. In paclitaxel DCBs, excipients influence coating cohesion, shear resistance, dissolution behavior, particle detachment, tissue adhesion, and washout.

Hydrophilic excipients can promote rapid release and acute transfer. Iopromide-based paclitaxel formulations, exemplified by the Paccocath/SeQuent Please lineage, established the benchmark rapid-transfer concept in coronary DCB therapy [1,26]. Flat-film experiments using porcine femoral tissue suggest that hydrophilic coating microstructure can affect acute transfer under the tested contact conditions [7], without establishing a clinical effect of that microstructure. Rapid release is beneficial only if enough coating survives delivery; otherwise, payload may be lost before lesion arrival or dispersed off target [4,21].

Urea-based and other hydrophilic systems support local deposition through formulation- and expansion-dependent interactions with the vessel surface [7,27]. Hydrophobic, plasticizer-based, butyryl-tri-hexyl citrate (BTHC)-containing, and resin-based formulations attempt to improve cohesion, reduce premature loss, and preserve drug availability until lesion contact [27]. These approaches should be interpreted as platform-level engineering strategies rather than interchangeable paclitaxel-class properties.

The AGENT platform provides a clinical anchor for the nominal-dose argument, but not direct proof of a microstructural mechanism. In AGENT IDE, a 2 µg/mm^2^ PCB reduced 1-year target-lesion failure (TLF) versus uncoated balloon angioplasty in coronary ISR (17.9% vs. 28.7%) [28]. Target-lesion revascularization (TLR), target-vessel myocardial infarction (MI), and definite/probable stent thrombosis were also lower (13.0% vs. 24.7%, 5.8% vs. 11.1%, and 0% vs. 3.2%, respectively). These data establish efficacy of the tested 2 µg/mm^2^ device against an uncoated balloon in coronary ISR. The trial neither compared paclitaxel doses nor isolated the coating-transfer mechanism.

Resin-based systems, including shellac–ammonium matrices, represent another approach to balancing stability and release. An in vitro and in vivo comparison of RESTORE with SeQuent Please assessed coating morphology, in vitro wash-off, acute tissue uptake, and porcine coronary histology [29]. Tissue paclitaxel was assayed 60 min after treatment, without a significant between-device uptake difference. This single acute measurement does not establish long-term retention or clinical superiority.

Adhesion switching summarizes the central excipient trade-off: a coating must be cohesive during tracking yet releasable during inflation. Fragile coatings may lose drug before lesion arrival, whereas overly stable coatings may release too little during balloon-vessel contact [4,8,21,30]. Robotic abrasion testing measures visible coating loss under artificial conditions [21]. It does not measure drug release into tissue or validate a coronary efficacy threshold.

## 5. Coating Morphology and Mechanical Stability

Coating morphology influences how much drug remains available at the lesion and how efficiently it is deposited during inflation. In paclitaxel systems, deposition methods and solvent–excipient combinations generate morphologies ranging from needle-like crystalline structures to smoother amorphous or semi-amorphous coatings, with consequences for cohesion, particulate loss, acute transfer, and tissue deposition [4,7,8,21].

Crystalline paclitaxel coatings may create a high local reservoir and facilitate particulate deposition, but brittle crystalline domains can fragment during handling, advancement, or expansion, leading to premature loss and particle shedding [4,21]. Amorphous or semi-amorphous coatings may improve mechanical integrity and reduce pre-inflation loss, depending on excipient and manufacturing process. Thus, the key variable is not crystallinity alone but the balance between payload preservation and triggered release [4,7,8,21]. At the same time, preclinical evidence suggests that particulate or crystalline paclitaxel deposition may favor tissue uptake and prolonged arterial retention after transfer. Granada et al. compared crystalline and amorphous paclitaxel formulations using serial tissue assays in rabbit abdominal aortas and a separate healing experiment in stented porcine coronaries [31]. These experiments inform formulation-specific hypotheses; the pharmacokinetic and healing measurements were obtained in different animal settings, without a human exposure–outcome analysis.

For sirolimus balloons, coating or carrier morphology is often essential for delivery success because the intended post-contact exposure depends on the formulation. The coating must provide particulate embedding, controlled release, carrier-mediated uptake, or reservoir-like persistence [14,15,16,17,18,19].

### 5.1. Resistance to Shear and Particle Loss

Mechanical testing can characterize coating abrasion or drug loss during simulated device handling [21]. Visible surface loss, recovered drug mass, tissue uptake, and clinical restenosis are different measurements. An abrasion result cannot quantify human coronary drug exposure or predict clinical restenosis.

### 5.2. Retention-Oriented Architecture in Sirolimus DCBs

Sirolimus DCB performance depends on whether the platform can generate sufficient tissue entry and sustain local exposure after balloon removal. Contemporary limus platforms use crystalline surface depots, nanoparticle encapsulation, phospholipid carriers, biodegradable micro-reservoirs, or porous pressure-driven infusion [14,15,16,17,18,19,20].

These architectures should be interpreted separately. Crystalline sirolimus coatings aim to deposit a temporary mural reservoir, whereas nanoparticle and phospholipid systems aim to improve transit stability, wall deposition, uptake, and delayed release. Poly(lactic-co-glycolic acid) (PLGA) micro-reservoir systems distribute drug-loaded biodegradable reservoirs across the vessel surface. Porous pressure-driven systems instead deliver liquid drug through micropores rather than relying on a conventional external coating [16,18,19,20].

Early clinical signals remain architecture-specific. In SABRE, the porous pressure-driven sirolimus system achieved 6-month late lumen loss of 0.31 ± 0.52 mm in 50 patients with coronary ISR, with 1-year target-lesion failure of 12.2% [18]. A microsphere-based sirolimus technology reported 6-month late lumen loss of 0.26 ± 0.45 mm in 56 de novo or restenotic lesions, with one late target-lesion revascularization [20]. These early, non-comparative studies support the feasibility of their respective delivery concepts but should not be pooled as generic sirolimus DCB evidence.

Randomized evidence is available for the crystalline SeQuent SCB platform. In a 70-patient de novo trial, SeQuent SCB was noninferior to SeQuent Please NEO for 6-month in-segment late lumen loss (0.11 ± 0.37 vs. 0.04 ± 0.39 mm), with few clinical events through 12 months [32]. The study supports the angiographic feasibility of this named platform after successful lesion preparation but was not powered for clinical outcomes. A single-patient report used a B. Braun crystalline sirolimus/BHT balloon with one 45 s inflation [33]. OCT showed hyperechoic particles with shadowing immediately after treatment; most had disappeared at 28 days, with a few residual particles. This is a morphological observation in one patient, without a chemical tissue drug assay. Human OCT descriptions of crystal-like material [33,34] do not, by appearance alone, establish drug identity, bioavailability, or a concentration–time profile.

In DES-ISR, a combined analysis of two randomized trials likewise showed noninferiority of SeQuent SCB to SeQuent Please/SeQuent Please NEO for 6-month in-segment late lumen loss (0.26 ± 0.60 vs. 0.25 ± 0.57 mm), without significant differences in 12-month clinical outcomes [35]. The conclusion remains restricted to the investigated crystalline platform because regional variability and differences among SCB technologies limit broader generalization. SIBLINT-ISR subsequently randomized 258 patients to SeQuent SCB or SeQuent Please NEO and reported noninferiority for nine-month in-segment late lumen loss (0.37 ± 0.48 versus 0.30 ± 0.38 mm; difference 0.07 mm, 95% CI −0.05 to 0.19; margin 0.20 mm) [36]. This angiographic result does not establish equivalent clinical-event risk or quantify human tissue exposure.

Biolimus-coated balloons also require device-specific interpretation. BIO ASCEND ISR randomized 280 patients and established nine-month angiographic noninferiority of BioAscend to SeQuent Please NEO (in-segment late lumen loss 0.23 ± 0.37 versus 0.25 ± 0.35 mm; difference −0.02 mm, 95% CI −0.12 to 0.07; margin 0.195 mm) [37]. REFORM tested a different investigational Biolimus A9 balloon in 202 patients and did not establish noninferiority to SeQuent Please for six-month in-segment diameter stenosis (43.3% versus 31.4%; 95% CI for the difference 4.9–18.8 percentage points; margin 12 percentage points) [38]. The devices, endpoints and margins differed. These trials cannot establish a biolimus class effect or explain their outcomes through coating mechanisms.

### 5.3. Coating Uniformity and Device-Level Consequences

Greater coating uniformity may improve the reproducibility of drug transfer. More homogeneous coatings may support more predictable balloon-tissue contact and release, whereas heterogeneous or brittle coatings may contribute to focal loss, particulate shedding, and underdosing. Efficacy depends on adequate exposure across the treated segment rather than a high average dose with poor spatial consistency [4,7,8,30].

Even within paclitaxel platforms, use of the same active drug does not guarantee an identical angiographic phenotype. In a head-to-head ISR comparison of two paclitaxel balloons with different coating strategies, IN.PACT Falcon showed lower in-segment late lumen loss than DIOR (−0.03 ± 0.43 vs. 0.36 ± 0.48 mm; *p* = 0.014) and higher distal fractional flow reserve (FFR) at follow-up (0.92 ± 0.07 vs. 0.84 ± 0.13). Neointimal volume decreased by 16% with IN.PACT Falcon but increased by 30% with DIOR [26]. This small, nonrandomized comparison of sequential cohorts measured angiographic, functional, and OCT outcomes. It did not isolate the coating variable or measure tissue drug exposure, so it cannot identify the mechanism underlying the observed difference.

Long-term comparison of AGENT PCB and SeQuent Please PCB provides additional device-level context within the paclitaxel family. In a 7-year DES-ISR analysis of 262 patients, risks of TLR, death, MI, and target-lesion thrombosis did not differ significantly between groups [39].

These findings require cautious interpretation because the SeQuent Please group was a historical control and important procedural differences were present: vessels and DCB diameters were larger in the AGENT group, lesion preparation was more frequent, and OCT was used in 23.2% of AGENT-treated lesions but in none of the SeQuent Please lesions. Accordingly, the study neither demonstrates platform heterogeneity nor establishes equivalence between the two paclitaxel devices. It primarily illustrates the limitations of non-randomized named-device comparisons and the need to account for lesion selection, preparation, imaging use, and temporal changes in practice [39].

A randomized comparison provides stronger evidence for a specific angiographic endpoint: in 204 patients with successfully prepared de novo lesions, the Genoss shellac/vitamin-E PCB was noninferior to the iopromide-based SeQuent Please NEO for six-month late lumen loss (0.06 ± 0.38 versus 0.09 ± 0.36 mm; upper one-sided 97.5% CI 0.08 mm; margin 0.15 mm) [40]. The primary per-protocol analysis and incomplete angiographic follow-up limit interpretation. This trial compares named devices but neither isolates an excipient effect nor establishes clinical equivalence.

## 6. Balloon-Vessel Contact Mechanics and Lesion Substrate

DCB efficacy also depends on lesion environment. Calcium burden, plaque composition, neointimal architecture, vessel geometry, lesion length, vessel size, tortuosity, and procedural injury influence apposition, contact-pressure distribution, penetration, tissue disruption, and early retention [1,6,22,23,25]. Lesion morphology is therefore an engineering constraint on drug delivery, not merely a clinical descriptor.

ISR provides a scaffolded environment in which acute recoil is constrained, but ISR remains biologically heterogeneous. Bare-metal stent restenosis and DES restenosis differ in neointimal composition, cellularity, lipid content, neoatherosclerosis, calcific change, inflammatory activity, and lesion distribution, all of which may influence transfer and restenosis suppression [1,26,28,35,39]. De novo lesions add recoil control, dissection management, and maintenance of acute luminal gain without a scaffold. Small-vessel disease is particularly sensitive to modest late lumen loss because small absolute reductions in lumen diameter can become clinically relevant restenosis [1,41,42,43,44,45,46].

Calcified lesions are a plausible example of substrate-limited DCB delivery because calcium can impair expansion, contact uniformity, and drug access to the arterial wall. The observational OCT/OFDI study described above linked calcium burden and post-PCI morphology with TLF, but did not establish a single drug-delivery mechanism [25].

Plaque-modifying strategies, including scoring balloons, cutting balloons, atherectomy, and intravascular lithotripsy, may improve acute lumen gain, calcium modification, and contact uniformity before DCB delivery [1,47,48]. The evidentiary status varies by lesion subset and study design; these approaches should be presented as lesion-preparation tools that may support DCB delivery rather than as universal requirements.

Bifurcations impose asymmetric wall contact, variable side-branch ostial coverage, altered flow, repeated device manipulation, and heterogeneous injury. DCB-BIF provides randomized evidence for a specific bifurcation setting: simple true bifurcation lesions treated with provisional main-vessel stenting in which the side branch remained severely compromised after main-vessel stenting and first proximal optimization. The trial included Medina 1,1,1; 0,1,1; or 1,0,1 lesions with main-vessel and side-branch reference diameters ≥2.5 mm, side-branch lesion length <10 mm, and ostial side-branch diameter stenosis ≥70% after main-vessel stenting. Patients were randomized to side-branch DCB or noncompliant balloon (NCB) angioplasty, and rescue side-branch stenting was reserved for type C dissection or Thrombolysis in Myocardial Infarction (TIMI) flow <3 [49].

In this setting, DCB-BIF randomized 784 patients to DCB (*n* = 391) or NCB (*n* = 393) treatment of the compromised side branch. The 1-year primary composite endpoint of cardiac death, target-vessel MI, or clinically driven TLR occurred in 7.2% versus 12.5% (hazard ratio (HR) 0.56, 95% confidence interval (CI) 0.35–0.88; *p* = 0.013), driven mainly by fewer myocardial infarctions. Procedural success, crossover to a 2-stent strategy, all-cause death, revascularization, and stent thrombosis did not differ significantly. These data support side-branch DCB angioplasty as a bifurcation-specific strategy, not as broad evidence for all de novo coronary DCB use [49].

Several limitations reinforce this narrow interpretation. Operators were not blinded, complex bifurcations were excluded, sirolimus-coated balloons were not tested, intravascular imaging and physiology were not used systematically, and high rates of periprocedural MI were described as early and of unclear clinical significance [49]. Bifurcation evidence should therefore remain tied to side-branch optimization after provisional main-vessel stenting.

Computational studies examine the effects of vessel geometry and plaque substrate on contact and transport [6,22,23]. These include an idealized femoral contact model [6], not a validated coronary simulation. Their estimates are conditional on the modeled assumptions and do not quantify coating loss or drug exposure in patients with tortuous, long, or ostial coronary lesions.

## 7. Technology Taxonomy of Contemporary Coronary DCB Platforms

Table 1 groups platforms by their reported delivery architecture. This is a technical description of where the drug is located and how delivery is intended to occur, independent of clinical efficacy or an evidence grade. Device-specific study designs and findings are reported separately in Table 2.

**Table 1:** Reported delivery architectures of coronary DCB platforms and experimental concepts.

Platform Family	Reported Drug Location Before Inflation	Proposed Delivery Mechanism	Proposed Persistence Strategy	Representative Examples
Rapid-transfer surface-coated PCB	Surface coating	Hydrophilic excipient-assisted dissolution and brief-contact deposition	Paclitaxel tissue partitioning, binding, and local particulate persistence	Paccocath/SeQuent Please lineage [1,50]
Engineering-variant surface-coated PCB	Modified surface coating	Platform-specific release, adhesion switching, and coating-vessel interaction	Platform-dependent tissue deposition and retention	AGENT, Pantera Lux, IN.PACT Falcon, RESTORE, DIOR [26,27,28,29,30,39,41,51]
Carrier-enabled surface-coated limus balloon	Surface coating with carrier support	Carrier-mediated uptake or particle-assisted transfer	Delayed local retention through carrier strategy	MagicTouch-type phospholipid/submicron systems [14,19,42,52]
Reservoir/eluting limus balloon	Drug-loaded micro-reservoirs or biodegradable carriers	Surface deposition with delayed release	Temporary local depot or sustained release	SELUTION SLR [14,53]
Porous-infusion limus system	Liquid drug within porous balloon	Pressure-assisted delivery through micropores	Depends on delivered mural load and formulation retention	SABRE/Virtue-type concepts [16,18]
Crystalline limus surface-depot balloon	Crystalline surface coating	Direct vessel-wall deposition of crystalline sirolimus	Temporary mural crystalline depot	SeQuent SCB [32,33,34,35]
Experimental hybrid or microgel systems	Experimental coating or carrier	Preclinical architecture-specific transfer	Experimental depot, microgel, or combination-drug persistence	Everolimus microgel and dual-drug nanoparticle concepts [54,55]

Note: This taxonomy describes reported or proposed delivery architectures and does not assign evidence maturity to a platform family. Clinical evidence is identified separately for each device, generation, indication, and study design in Table 2 and Supplementary Table S1. Findings from one device are not assigned to another device sharing its drug or architectural category. DCB, drug-coated balloon; PCB, paclitaxel-coated balloon; PLGA, poly(lactic-co-glycolic acid); SCB, sirolimus-coated balloon.

First, benchmark rapid-transfer paclitaxel platforms, represented by the Paccocath/SeQuent Please lineage, address brief-contact therapy through rapid coating dissolution, early tissue transfer, hydrophobic sequestration, and durable antiproliferative effect [1,26,50]. Separate coronary studies report angiographic changes and clinical outcomes. Those endpoints do not verify the preceding delivery mechanisms in patients.

Second, modified paclitaxel platforms retain paclitaxel but alter excipient composition, coating architecture, dose density, or surface morphology. Urea-based, hydrophilic, hydrophobic, plasticizer-based, and resin-based strategies test whether handling stability, washout resistance, transfer efficiency, and spatial uniformity can be optimized while preserving paclitaxel pharmacology [8,27,28,29,30,39]. Their readouts should be interpreted as platform-specific rather than proof that all paclitaxel balloons behave similarly. Representative named platforms and their engineering features are summarized in Table 2.

**Table 2:** Selected named coronary DCB platforms and evidence relevant to brief-contact delivery.

Platform/Device	Manufacturer/Owner	Drug and Nominal Surface Dose	Reported Coating/Carrier Architecture	Direct PK Measurements in Cited Sources	Human Evidence and Setting	Interpretation
SeQuent Please	B. Braun	Paclitaxel; 3.0 µg/mm^2^ [1,50]	Paclitaxel–iopromide hydrophilic matrix [1,50]	Porcine coronary paclitaxel assayed at 60 min versus RESTORE [29]. No human coronary tissue assay in ref. [50].	Randomized ISR trials and long-term follow-up of the earlier PACCOCATH platform [50]; lineage evidence is identified explicitly.	Benchmark rapid-transfer PCB; conclusions apply to the iopromide lineage.
SeQuent Please NEO	B. Braun	Paclitaxel; 3.0 µg/mm^2^ [32,35,42]	Newer-generation iopromide/paclitaxel matrix [32,35,42]	Not measured in the cited human studies [32,35,42].	Randomized comparator in de novo/small-vessel and ISR trials [32,35,42].	Generation-specific evidence should be distinguished from SeQuent Please.
AGENT	Boston Scientific	Paclitaxel; 2.0 µg/mm^2^ [28,39]	ATBC (TransPax) excipient coating [28,39]	Porcine coronary and downstream myocardial paclitaxel concentrations at 28 days [14]. No human arterial tissue assay in the cited trials.	AGENT IDE randomized ISR trial [28]; separate historical-control DES-ISR comparison [39].	Clinical efficacy of a 2 µg/mm^2^ device versus an uncoated balloon does not establish a dose–response or coating mechanism.
Pantera Lux	Teleflex (formerly BIOTRONIK)	Paclitaxel; 3.0 µg/mm^2^ [27,51]	BTHC matrix [27,51]	Porcine coronary paclitaxel assays for a BTHC formulation [27]; identity with the current commercial generation is not established here. No human tissue assay.	Prospective observational DELUX registry, with mixed coronary indications [51].	BTHC-based platform; current ownership is distinguished from the manufacturer named in earlier studies.
IN.PACT Falcon	Medtronic	Paclitaxel; 3.0 µg/mm^2^ [26]	Urea-based FreePac matrix [26]	Not measured in the cited coronary comparison [26].	Small comparative coronary ISR study versus DIOR [26].	Useful same-drug/different-coating comparison; clinical readouts are not direct pharmacokinetic measurements.
RESTORE	Cardionovum	Paclitaxel; 3.0 µg/mm^2^ [29,41]	SAFEPAX shellac–ammonium matrix [29,41]	Porcine coronary tissue paclitaxel assayed at 60 min [29]. Single acute time point; no human coronary tissue assay in ref. [41].	RESTORE SVD China randomized small-vessel trial versus DES [41].	Preclinical coating findings and clinical outcome evidence are presented separately.
DIOR	Eurocor (historical)	Paclitaxel; 3.0 µg/mm^2^ [26]	Shellac-based coating lineage [26]	Not measured in the cited coronary comparison [26].	Small comparative coronary ISR study versus IN.PACT Falcon [26].	Do not transfer findings to a different DIOR generation without confirming device identity.
MagicTouch SCB	Concept Medical	Sirolimus; 1.27 µg/mm^2^ [14,19,42,52]	Phospholipid/submicron nanocarrier technology [19,42]	Porcine coronary and myocardial sirolimus concentrations at 28 days [14]. Developmental nanocarrier work [19] is separate; no human tissue assay.	TRANSFORM-I randomized small-vessel trial [42]; OCT substudy of that trial [52], not independent replication.	TRANSFORM I did not establish noninferiority for net lumen gain; conclusions remain device-specific.
SELUTION SLR	Cordis (MedAlliance technology)	Sirolimus; 1.0 µg/mm^2^ [53]	PLGA micro-reservoirs in a phospholipid cell-adherent matrix [14,53]	Porcine coronary and myocardial sirolimus concentrations at 28 days [14]. No human coronary tissue assay in ref. [53].	SELUTION DeNovo strategy trial [53]: primary ITT noninferiority, not confirmed in PP analysis; provisional DES in 20.7%. SELUTION4ISR [56]: primary PP noninferiority versus mixed usual care; prespecified single-layer comparison versus repeat DES did not meet noninferiority.	SELUTION DeNovo evaluates a provisional-stenting strategy. SELUTION4ISR evaluates ISR against mixed usual care. Neither establishes DES equivalence or a class-wide SCB effect.
SeQuent SCB	B. Braun (InnoRa coating technology)	Sirolimus; 4.0 µg/mm^2^ [32,35]	Highly crystalline sirolimus with BHT excipient [32,35]	No human tissue drug assay in the cited trials [32,35] or single-patient OCT report [33]. OCT morphology is separate from chemical drug measurement.	Randomized de novo and DES-ISR angiographic trials [32,35,36]. SIBLINT-ISR reports device-specific angiographic noninferiority. Related B. Braun crystalline sirolimus/BHT balloon described in one OCT case [33].	OCT alone cannot verify drug identity, concentration, or bioavailability. Angiographic efficacy and PK are separate questions.

Notes: Nominal dose denotes drug mass per nominal expanded balloon surface area (µg/mm^2^), not the dose delivered to or retained in tissue. Architecture descriptions are manufacturer- or trial-reported unless an experimental source is specified. The cited human studies do not provide direct coronary tissue drug concentrations. OCT appearances, histology, angiographic endpoints, and clinical events are not pharmacokinetic measurements. Preclinical assays are attributed only to the tested device or formulation and model; related formulations are not assumed to be identical commercial generations. Manufacturer and historical ownership names identify the cited devices; jurisdiction-specific availability was not assessed. Abbreviations: ATBC, acetyl tributyl citrate; BHT, butylated hydroxytoluene; BTHC, butyryl-tri-hexyl citrate; DCB, drug-coated balloon; DES, drug-eluting stent; FFR, fractional flow reserve; ISR, in-stent restenosis; ITT, intention-to-treat; IVUS, intravascular ultrasound; OCT, optical coherence tomography; PCB, paclitaxel-coated balloon; PLGA, poly(lactic-co-glycolic acid); SCB, sirolimus-coated balloon.

Third, carrier-enabled limus platforms use engineered uptake support or pressure-assisted delivery, whereas reservoir-oriented platforms seek sustained exposure through temporary depots [14,15,16,17,18,19,20]. Despite a shared limus payload, these systems differ in pre-inflation drug location, interface crossing, and post-removal exposure and should therefore be evaluated separately.

## 8. Angiographic and Imaging Outcomes

Angiographic, imaging, and clinical endpoints characterize vessel response and treatment outcomes. They do not measure drug transfer, concentration, or retention. Table 3 specifies what each type of measurement can establish and where an additional experiment would be needed. The following studies are therefore interpreted within their own devices, populations, comparators, and endpoints, without attributing their findings to delivery features measured in unrelated experiments.

**Table 3:** Measurements and limits of inference in DCB studies.

Measurement Domain	Representative Sources	What Is Measured	Permissible Inference	What Is Not Established
Coating and handling tests	Kempin [4]; Heinrich [21]; Xia [29]	Visible coating change, recovered material, wash-off or particle characteristics under a specified test.	Technical behavior of the tested product or formulation in that test.	Human coronary tissue exposure, clinical restenosis, or a device ranking based on incompatible test methods.
Computational transport and contact models	Escuer [5]; Stratakos [6]; Anbalakan [22,23]	Calculated contact pressure, drug transport or retention conditional on model inputs.	Predictions and sensitivity to the chosen assumptions.	Direct tissue drug measurements or clinical efficacy; estimates require external validation.
Direct preclinical drug measurements	Kawai [14]; Clever [15]; Granada [16]; Kolachalama [17]; Lemos [19]; Xia [29]; Granada [31]	Drug amount/concentration at specified sites and times; distinguish single-time-point assays from serial profiles.	Exposure in the tested formulation, species, vascular bed and protocol.	A therapeutic concentration threshold or pharmacokinetic mechanism for a different device or human trial.
Peripheral tracking study	Tscheuschner [24]	Drug loss and distribution during peripheral PearlFlow delivery; experimental components use different device sizes.	Findings confined to the tested peripheral setup.	Coronary delivery fractions, tissue exposure, safety or efficacy.
Histology and deposit imaging	Xia [29]; Jiménez Díaz [33,34]	Porcine vascular histology or human OCT appearances, respectively.	Morphological description in the observed tissue or lesion.	Chemical drug identity, active tissue concentration, release kinetics, or proof of pharmacological action.
Angiography and serial IVUS/OCT	TRANSFORM-I [42,52]; remodeling cohorts [25,57,58,59,60]	Lumen dimensions, vessel/plaque geometry, dissection or follow-up morphology.	Observed treatment differences or associations appropriate to the study design.	Tissue PK or mediation of remodeling by a coating, excipient or retention feature.
Clinical events	AGENT IDE [28]; REC-CAGEFREE I [43]; DCB-BIF [49]; SELUTION DeNovo [53]	Prespecified clinical endpoints in defined populations and treatment strategies.	Comparative clinical effect within the design and analysis population.	Which engineering feature caused that effect, or clinical performance of an untested device.

Note: Rows define separate measurement domains, not sequential steps in a validated mechanism. A chemical tissue assay is a direct drug measurement; histology and OCT are not substitutes. Causal attribution requires aligned measurements and suitable controls within the same platform and experimental or clinical setting. OCT, optical coherence tomography; IVUS, intravascular ultrasound; PK, pharmacokinetics;

### 8.1. Late Lumen Loss, Binary Restenosis, and Remodeling

Late lumen loss is the reduction in minimum lumen diameter from immediately after the procedure to follow-up. Neointimal growth, recoil, and remodeling can affect this endpoint. Low or negative late lumen loss does not quantify tissue drug exposure, and binary restenosis does not identify inadequate drug transfer or retention as its cause. Interpretation depends on the angiographic analysis segment, timing, and population; in-balloon/in-stent and in-segment measurements are not interchangeable across trials [1,10,25,32,42,57,58].

Late lumen enlargement is a measurable remodeling phenotype in selected paclitaxel DCB cohorts. Kleber et al. reported an increase in minimum lumen diameter from 1.75 ± 0.55 to 1.91 ± 0.55 mm at 4.1 ± 2.1 months in 58 de novo lesions, with 69% of patients showing net enlargement [59]. Intravascular ultrasound (IVUS) data by Yamamoto et al. identified late lumen enlargement in 74.1% of 54 lesions at 9 months and linked it to vessel enlargement and plaque regression. OCT studies have also characterized this phenotype; an OCT/OFDI analysis found late lumen enlargement in 40.7% of 108 lesions and associated it with layered plaque and extensive medial dissection [57,58,60]. These imaging observations should be interpreted as observational remodeling phenotypes, not as proof of a single drug-mediated pathway.

Foundational de novo small-vessel trials provide important angiographic context. In BELLO, 182 patients with small-vessel lesions were randomized to a paclitaxel drug-eluting balloon or paclitaxel-eluting stent. In-balloon/in-stent late loss was lower with the DCB strategy (0.08 ± 0.38 vs. 0.29 ± 0.44 mm; difference −0.21 mm, 95% CI −0.34 to −0.09; *p* for noninferiority < 0.001; *p* for superiority = 0.001). At 6 months, restenosis was 8.9% versus 14.1%, TLR was 4.4% versus 7.6%, and major adverse cardiac events (MACE) occurred in 7.8% versus 13.2% [44]. In RESTORE SVD China, 230 patients with de novo small-vessel disease were randomized to RESTORE DCB or RESOLUTE Integrity DES. The 9-month in-segment percentage diameter stenosis was 29.6 ± 2.0% versus 24.1 ± 2.0%, meeting the prespecified noninferiority criterion; 1-year TLF was 4.4% versus 2.6% [41].

Platform-specific remodeling is visible in randomized small-vessel data. In TRANSFORM-I, MagicTouch SCB did not meet noninferiority to SeQuent Please NEO PCB for 6-month angiographic net lumen gain (0.25 ± 0.40 vs. 0.48 ± 0.37 mm; absolute difference −0.23 mm, 95% CI −0.37 to −0.09). Binary restenosis was higher after SCB (32.8% vs. 12.5%), and late lumen enlargement was less frequent (30.0% vs. 53.7%) [42]. In the OCT substudy, lumen volume decreased after SCB (97.35 ± 71.09 to 87.96 ± 61.48 mm^3^; *p* = 0.03) but not after PCB (69.67 ± 38.24 to 71.64 ± 42.22 mm^3^; *p* = 0.64) [52]. The OCT substudy was small, and a significant within-group change in one arm but not the other does not establish a between-group difference. Neither the randomized angiographic comparison nor the OCT substudy measured tissue drug exposure [42,52].

By contrast, the crystalline SeQuent SCB trial reported angiographic noninferiority to SeQuent Please NEO after successful lesion preparation [32]. Together, these trials illustrate that results from one sirolimus platform cannot be generalized to another.

### 8.2. Dissection Biology and Lesion Preparation

Coronary dissection after balloon angioplasty reflects mechanically induced cleavage of the vessel wall, usually along intimal-medial planes. Determinants include balloon sizing and pressure, plaque composition, calcium geometry, lesion eccentricity, vessel compliance, and stress amplification at compliant-rigid interfaces. In DCB angioplasty, controlled non-flow-limiting dissection should not automatically be considered procedural failure, but severe dissection with impaired flow, extensive recoil, vessel closure risk, or instability remains an indication for bailout stenting [1,61].

Lesion preparation aims to achieve an acceptable acute mechanical result. Preparation can improve acute luminal gain, compliance, and balloon expansion; any resulting change in drug transfer requires direct measurement. Scoring and cutting balloons can create controlled plaque modification and intimal micro-incisions; atherectomy or intravascular lithotripsy may improve compliance and expansion in calcified lesions [1,47,48].

The NATURE exploratory randomized trial compared Wolverine cutting-balloon with standard-balloon preparation before an AGENT-based DCB strategy. Of 210 randomized patients, 201 entered the reported intention-to-treat analysis. The primary composite of optimal lesion preparation was not significantly improved (35.6% versus 30.0%; adjusted RR 1.19, 95% CI 0.87–1.65), despite modest acute angiographic advantages [62]. The pilot had no formal power calculation for superiority and reported acute and 30-day outcomes. It does not demonstrate increased drug uptake, support deliberate medial injury or establish long-term clinical benefit.

The association between medial dissection and favorable remodeling should be interpreted carefully. In studies of late lumen enlargement, greater dissection index or extensive medial dissection was associated with subsequent lumen enlargement after paclitaxel DCB treatment [57,58], while absence of post-PCI medial dissection predicted higher TLF in an observational de novo DCB PCI cohort [25]. These findings do not justify deliberate creation of deep dissections. Post-DCB medial dissection may mark effective lesion preparation, plaque modification, and compliance gain in selected cohorts, but current evidence is still observational and should not be interpreted as a procedural target.

For clinical practice and trial interpretation, the relevant procedural objective remains adequate lesion preparation with TIMI 3 flow, acceptable residual stenosis and recoil, and no angiographic or imaging evidence of flow-limiting dissection or vessel instability requiring bailout stenting. The cited limus-balloon trials and imaging studies do not establish that deliberately increasing wall disruption improves human tissue drug exposure [32,42,52]. Bailout stenting should therefore be discussed as a safety-driven procedural outcome rather than as an intended feature of DCB angioplasty.

### 8.3. Clinical Outcomes by Device and Lesion Setting

Clinical outcome data are evaluated independently of mechanistic hypotheses. Randomized trials, registries, imaging studies, and meta-analyses occupy different evidentiary layers. Clinical outcomes test whether a treatment strategy improves patient- or lesion-level events; they do not by themselves identify the coating, transfer, retention, or tissue pharmacokinetic mechanism responsible for the result. Conversely, bench, computational, and porcine studies can identify delivery vulnerabilities but do not establish clinical efficacy.

Coronary ISR has randomized and long-term follow-up evidence for specific paclitaxel platforms. Long-term follow-up of the PACCOCATH-ISR I/II trials compared paclitaxel-coated balloon therapy with uncoated balloon angioplasty in 108 patients with ISR. At 5.4 ± 1.2 years, the paclitaxel-coated balloon strategy was associated with lower MACE (27.8% vs. 59.3%) and TLR (9.3% vs. 38.9%) than control angioplasty [50]. These data provide an important historical coronary anchor for the Paccocath/SeQuent Please lineage.

De novo small-vessel disease has a separate evidence base and should not be judged solely by broad all-comer de novo trials. In BASKET-SMALL 2, 758 patients with de novo lesions in vessels <3 mm were randomized after successful predilation to DCB or DES. At 12 months, MACE was 7.5% vs. 7.3% in the full-analysis population (HR 0.97, 95% CI 0.58–1.64), and noninferiority was met in the per-protocol population [45]. Three-year follow-up showed sustained similarity, with Kaplan–Meier MACE 15% vs. 15% and target-vessel revascularization 9% vs. 9% [46]. Together with BELLO and RESTORE SVD China, these trials support selected DCB use in de novo small-vessel disease when lesion preparation is adequate [41,44,45,46].

The broader de novo setting is more complex. REC-CAGEFREE I was an open-label, randomized, noninferiority trial at 43 Chinese centers that included 2272 patients with de novo noncomplex coronary lesions after successful predilation. Patients were assigned to paclitaxel-coated balloon angioplasty with rescue stenting or intended second-generation thin-strut sirolimus DES; rescue DES was required in 9.4% of the DCB group. At 24 months, the device-oriented composite endpoint was higher with the DCB strategy (6.4% vs. 3.4%). The absolute risk difference was 3.04%, and the upper one-sided 95% CI boundary was 4.52%, so the prespecified noninferiority criterion was not met [43]. This result is a negative randomized signal for broad de novo noncomplex coronary use, not a refutation of selected small-vessel, bifurcation, or lesion-prepared DCB strategies.

SELUTION DeNovo randomized 3341 patients before PCI to a SELUTION SLR sirolimus-eluting balloon strategy with provisional DES or systematic DES; 3323 patients were included in the primary analysis, and bailout stenting was required in 20.7% of patients assigned to the balloon strategy. In the primary intention-to-treat analysis, 1-year target-vessel failure occurred in 5.3% versus 4.4%, meeting the prespecified noninferiority criterion. However, the per-protocol sensitivity analysis did not confirm noninferiority, and clinically driven target-vessel revascularization was more frequent with the SELUTION strategy (3.3% vs. 2.1%) [53]. The trial therefore provides a statistically qualified signal for the SELUTION SLR minimal-stenting strategy rather than evidence of equivalence to DES or a class-wide reversal of REC-CAGEFREE I.

The historical AGENT-SeQuent Please comparison was confounded and cannot establish either heterogeneity or equivalence between the devices [39].

For limus platforms, the combined randomized DES-ISR analysis reported angiographic noninferiority of SeQuent SCB to SeQuent Please and no significant difference in 12-month clinical outcomes [35]. This supports the investigated crystalline platform but does not extend to SCBs with different coating technologies or drug loads. An ISR-focused head-to-head meta-analysis likewise reported no statistically significant difference in pooled clinical outcomes between limus- and paclitaxel-coated balloons; a nonsignificant pooled difference does not establish equivalence or noninferiority, and this conclusion should not be extrapolated to de novo lesions or heterogeneous angiographic surrogates [63].

SELUTION4ISR randomized 418 patients with ISR and tested SELUTION SLR against operator-preselected usual care comprising repeat DES or plain balloon angioplasty. In the primary per-protocol population of 390 patients, one-year target-lesion failure was 16.2% versus 14.5% (difference 1.7 percentage points, 95% Bayesian credible interval −5.5 to 8.9), meeting the 10-percentage-point noninferiority margin. The prespecified sequential comparison with repeat DES in single-layer ISR did not establish noninferiority [56]. The primary result therefore applies to the mixed-control strategy and cannot be interpreted as equivalence to repeat DES or evidence of a delivery mechanism.

Pooled paclitaxel-versus-limus comparisons can obscure differences in excipient chemistry, coating morphology, drug dose, carrier design, reservoir strategy, indication, and trial design [32,35,42,64,65]. In Sedhom et al.’s meta-analysis of six randomized trials including 821 patients, clinically driven TLR did not differ significantly between limus and paclitaxel DCBs (10.3% vs. 7.8%; risk ratio (RR) 1.32, 95% CI 0.84–2.08). Angiographic surrogates were less favorable with limus DCBs: binary restenosis RR 1.89 (95% CI 1.14–3.12), late lumen loss mean difference 0.16 mm (95% CI 0.03–0.28), and follow-up minimum lumen diameter mean difference −0.12 mm (95% CI −0.22 to −0.02) [65]. These pooled endpoints do not measure delivery or retention. They cannot identify a pharmacokinetic mechanism or rank individual platforms, particularly when lesion settings and devices differ.

DCB-BIF also provided lesion-specific randomized support for side-branch DCB angioplasty after provisional main-vessel stenting, with the observed benefit driven mainly by MI and limited to the selected simple true-bifurcation population [49].

Other comparative syntheses, including Shin 2024, should be interpreted cautiously at the device level because pooled analyses combine heterogeneous platforms, lesion settings, and endpoints [64]. Clinical setting determines the applicability of each trial result. ISR studies and de novo studies address different lesion environments. Selected and adequately prepared de novo small-vessel lesions have supportive randomized evidence, whereas broader de novo use has produced mixed platform-specific results, including REC-CAGEFREE I and SELUTION DeNovo. Bifurcation treatment addresses a different mechanical problem related to side-branch optimization and avoidance of additional metal. These settings should not be pooled into a single DCB class narrative.

### 8.4. Boundaries of Mechanistic Inference

A clinical result for one DCB cannot be mechanistically explained by a tissue assay obtained with another platform, species, vascular bed, or inflation protocol. Likewise, an OCT finding cannot establish tissue drug concentration. Matched formulation experiments can investigate selected delivery relationships under their own experimental conditions [15,16,17,31]. Where exposure and vascular response are measured in different species or vascular beds, the link remains indirect even within the same experimental program. These experiments do not establish mediation of human clinical outcomes. The evidence considered here therefore consists of distinct measurement domains with incomplete connections, rather than a demonstrated delivery-to-outcome continuum.

A porcine coronary experimental programme illustrates the distinction between drug recovery and efficacy. Haase et al. measured acute arterial transfer for selected alternative payloads, yet none of seven tested non-paclitaxel coatings significantly reduced four-week late lumen loss against pooled uncoated controls [66]. Experiments conducted over several years used different formulations and doses; some transfer measurements were not reported. These limitations prevent separation of drug, formulation and model effects. Detectable arterial drug should therefore not be treated as an antiproliferative efficacy endpoint.

## 9. Future Engineering Directions and Evidence Gaps

Future DCB technologies should be developed around coupled mechanical-pharmacokinetic efficacy. The objective is not simply higher balloon drug load, but better payload preservation, triggered release, wall adhesion, reduced off-target loss, temporary tissue-reservoir formation, and lesion-specific exposure [6,7,8,22,67]. Paclitaxel development will likely focus on improved coating cohesion, lower particle loss, more homogeneous deposition, reduced downstream dispersion, and efficacy at lower nominal doses. Limus development will likely focus on sustaining exposure through crystalline optimization, nanocarriers, phospholipid systems, porous infusion, PLGA micro-reservoirs, or related depot strategies [14,15,16,17,18,19,20].

Experimental future concepts, including microgel-coated balloons, dual-drug nanoparticle systems, and hybrid architectures, should be presented as preclinical or translational engineering directions rather than contemporary coronary clinical platforms [54,55]. They are relevant to future platform design but should not be grouped with devices supported by randomized coronary evidence.

Computational modeling can support device design and lesion selection by integrating contact pressure, curvature, plaque substrate, binding kinetics, washout, and coating loss [5,6,22,23,67]. However, computational results should be treated as mechanistic filters and hypothesis generators, not as clinical outcome evidence. Imaging should be incorporated into platform evaluation because calcium, bifurcations, long lesions, and ISR subtypes impose different constraints on expansion, contact, and drug distribution [1,25,47,48,49].

Several gaps remain despite the growing randomized evidence base. First, head-to-head comparisons between coronary DCB platforms remain limited, and broad drug-family comparisons obscure differences in excipient chemistry, coating morphology, dose, carrier design, and retention strategy [14,32,35,39,42,64,65]. Recent studies support specific platforms, including AGENT PCB, SeQuent Please PCB, SeQuent SCB, SELUTION SLR, and side-branch DCB use in DCB-BIF, but the findings do not extend to all paclitaxel or limus balloons [32,35,36,49,53,56]. Second, mechanistic endpoints are not standardized. Studies measure visible coating loss, drug mass loss, acute transfer, tissue retention, particle shedding, transmural distribution, and downstream drug effects differently, limiting cross-platform comparison [4,7,8,14,21,23,67].

Third, extrapolation across experimental systems is imperfect. Healthy porcine coronaries, bench abrasion models, ex vivo tracking circuits, and in silico simulations isolate important mechanisms but cannot fully reproduce calcified, lipid-rich, restenotic, bifurcating, or tortuous human coronary lesions. Fourth, lesion-stratified data remain sparse. Plaque morphology, calcification, vessel size, bifurcation anatomy, ISR subtype, dissection pattern, and lesion preparation likely influence DCB efficacy but are inconsistently reported [1,6,22,23,25,47,48,49]. Fifth, the designs, populations, and follow-up of studies differ across limus platforms, and findings from one architecture may not transfer to another [14,15,16,17,18,19,20,32,42]. Finally, the prognostic value of OCT/IVUS remodeling patterns across DCB platforms remains incompletely defined [25,52,57,58,59,60]. Future studies should link acute lesion preparation, delivery mechanics, follow-up remodeling, and clinical outcomes at the named-platform level.

## 10. Conclusions

Coronary DCBs are brief-contact drug-delivery platforms. Devices differ in drug, excipient or carrier, coating architecture, and intended delivery and retention strategy. Technical differences require device-specific evaluation; clinical results do not by themselves identify which feature accounts for an outcome. Nominal surface dose and drug family alone are therefore insufficient descriptors, and findings should be interpreted at the level of the named platform and lesion setting.

Clinical evidence is most established for coronary ISR and is supportive in selected de novo small-vessel disease, whereas broader de novo use has produced mixed, platform- and strategy-specific randomized results. Bifurcation treatment represents a distinct lesion-specific application, and evidence in calcified and other complex lesions is predominantly observational. Paclitaxel- and limus-based balloons each comprise heterogeneous technologies, so mechanistic, imaging, and clinical findings should not be generalized across devices.

Future studies should directly compare named platforms using standardized measures of coating loss, drug transfer and tissue exposure, imaging-defined response, and clinically relevant outcomes. Linking engineering performance to lesion-specific pharmacokinetics and long-term clinical durability is necessary to determine which platforms perform reliably in specific coronary settings.

## Data Availability

Evidence is available in the cited publications. Supplementary Table S1 provides the appraisal of cited reports. Supplementary Methods S1 provide the exact revision-stage PubMed queries.

## Supplementary Materials

The following supporting information can be downloaded at https://www.mdpi.com/article/10.3390/medicina62101932/s1. Supplementary Table S1: Study level critical appraisal of the cited DCB literature; Supplementary Methods S1: Search and evidence assessment during revision.
