# Supplementary material for: Coronary Drug-Coated Balloons as Brief-Contact Drug-Delivery Platforms: Engineering, Tissue Pharmacokinetics, and Angiographic/Imaging Response
Source: Medicina (Kaunas). 2026 Oct 7;62(10):1932. doi: 10.3390/medicina62101932 (PMC13706780; doi:10.3390/medicina62101932)
Supplement: Supplementary file 1 [file medicina-62-01932-s001.zip › medicina-4505945-supplementary.pdf]

Supplementary Table S1

Study level critical appraisal of the cited DCB literature

Reference numbers correspond to the accompanying manuscript. This table appraises the original 60 cited reports and seven additional reports identified during revision, including contextual reviews and consensus documents. It identifies what each source can support. The assessment is descriptive and claim-specific; it is not a validated risk-of-bias score. The separate revision-stage search and evidence map are documented in Supplementary Methods and Data S1.

Source verification was performed during this revision on 9 September 2026.

Report	Design and sample	Measured endpoints	Study-specific appraisal	Inference
[1] Fezzi et al., 2025	DCB-ARC position statement on indications	Synthesis or recommendations, not a new primary experiment.	Retained for clinical context. Not assigned a primary-study quality rating or counted as independent outcome evidence.	Primary experimental or comparative clinical claims require the underlying original studies.
[2] Marchetta et al., 2025	Narrative review of coronary clinical applications	Synthesis or recommendations, not a new primary experiment.	Retained for clinical context. Not assigned a primary-study quality rating or counted as independent outcome evidence.	Primary experimental or comparative clinical claims require the underlying original studies.
[3] Camaj et al., 2025	Clinical review of coronary DCB therapy	Synthesis or recommendations, not a new primary experiment.	Retained for clinical context. Not assigned a primary-study quality rating or counted as independent outcome evidence.	Primary experimental or comparative clinical claims require the underlying original studies.
[4] Kempin et al., 2015	Comparative bench study; five paclitaxel coating formulations; perfused simulated coronary pathway and hydrogel acceptor Five formulations	Paclitaxel lost during passage, transferred to hydrogel, and retained on balloon; coating microscopy.	Stage-specific mass accounting and formulation comparisons address transfer efficiency. Hydrogel is a tissue surrogate and lacks disease, perfusion-mediated retention and healing.	Supports formulation-dependent simulated loss and transfer; percentages are protocol-specific and do not establish coronary bioavailability or clinical superiority.
[5] Escuer et al., 2022	2D axisymmetric computational DCB/DES drug-transport model; multilayer artery with diffusion, advection and two binding phases	Predicted total tissue drug content and specific binding-site saturation under alternative drugs, delivery systems and inflation conditions.	Explicit transport/binding model permits controlled comparison of assumptions. Outputs depend on idealized geometry, assigned material/kinetic parameters and boundary conditions.	Predicted drug content and binding saturation are mechanistic surrogates; they do not demonstrate patient safety, efficacy or equivalence to DES.

Report	Design and sample	Measured endpoints	Study-specific appraisal	Inference
[6] Stratakos et al., 2023	Finite-element balloon-folding/inflation model within idealized healthy femoral arteries	Computed balloon–vessel contact-pressure distribution. No direct drug-transfer or clinical endpoint was measured.	Mesh sensitivity and explicit variation of folding, wall thickness, friction and vessel properties strengthen mechanical analysis. Idealized homogeneous cylinders, assumed balloon thickness and limited parameter space restrict anatomical realism; contact-pressure predictions lacked direct in vivo measurement.	Explains possible contact heterogeneity; does not validate an inflation pressure or oversizing prescription for coronary DCB therapy.
[7] Shazly et al., 2024	Bench/ex vivo porcine femoral artery study plus illustrative finite-element model; paclitaxel with urea or PEG on flat Nylon-12 films<br>Five coating variants per excipient; acute transfer experiments N=6 repeat measures per condition	SEM coating morphology, LC–MS acute paclitaxel transfer and modeled transient drug levels.	Controlled compositional/preparation variation and quantitative transfer assays link morphology with delivery. One-dimensional flat contact omits full balloon deployment; healthy tissue and limited variants constrain extrapolation. Model omits binding/unbinding and predicts total transient levels.	Supports a coating-aggregation/acute-transfer association under specified conditions; does not establish durable retention, embolic safety or human efficacy.
[8] Chang et al., 2019	Bench/ex vivo porcine femoral artery and cell study with Hertz contact model; experimental paclitaxel/urea and paclitaxel/shellac coatings	SEM morphology, force–displacement response, acute tissue drug transfer and endothelial-cell viability.	Combines orthogonal mechanical, chemical and cell measurements. Flat uniaxial contact and assumed linear elasticity do not reproduce deployment; ex vivo tissue viability and absent physiological washout limit retention inference. Adhesive and cohesive failure were not separately isolated.	Supports excipient-dependent contact and transfer behavior; cell-exposure toxicity cannot be treated as clinical endothelial harm or comparative device safety.
[9] Marchetta et al., 2026	Narrative mechanistic review	Synthesis of published engineering, pharmacology and clinical studies.	Contextual secondary source; not independently counted as primary mechanistic evidence or assigned a primary-study quality rating.	Comparative pharmacology and platform claims require support from the underlying primary studies.
[10] Fezzi et al., 2025	DCB Academic Research Consortium consensus on definitions and endpoints	Consensus definitions and recommendations for study design, imaging endpoints and follow-up.	Contextual methodological source, not an original efficacy or pharmacokinetic study.	Use to standardize terminology and endpoints, not to supply comparative mechanistic evidence.

Report	Design and sample	Measured endpoints	Study-specific appraisal	Inference
[11] Clever et al., 2011	Human EPC/hCASMC culture experiments plus randomized porcine coronary stent-healing experiment; paclitaxel balloon+BMS versus BMS and first-generation DES<br>Cell experiments at least triplicate; EPC obtained from one volunteer; 19 pigs and 38 stented vessels	Cell density/proliferation, migration and apoptosis after timed drug±iopromide exposure; 3-day histological endothelialization.	Two blinded investigators counted cell-density/apoptosis outcomes; animal treatments were randomized. Single-donor EPCs and brief follow-up restrict generalization. Animal comparison combines drug, carrier and scaffold/delivery differences; it is not a head-to-head comparison of current SCBs and PCBs.	Establishes concentration/time/cell-dependent responses; cannot prove an intrinsic clinical safety or efficacy advantage of one drug class.
[12] De Labriolle et al., 2009	Narrative review of non-stent local drug delivery	Historical preclinical and clinical synthesis.	Contextual secondary source; no primary study-level quality rating is applicable.	Supports historical rationale only; contemporary platform claims need direct primary evidence.
[13] Sabaté, 2022	Editorial/commentary	Interpretation of comparative sirolimus/paclitaxel evidence; no original outcomes assessed.	Contextual source, not an independent primary study.	Do not treat editorial interpretation as measured pharmacokinetic or comparative efficacy evidence.
[14] Kawai et al., 2025	Porcine coronary comparison of MagicTouch SCB, SELUTION SCB, AGENT PCB and POBA; 28-day assessment<br>10 pigs; 28 arteries, seven per treatment; histology in six pigs and pharmacokinetics in four	Arterial histology, tissue drug concentration and downstream myocardial emboli/injury.	Direct platform comparison with POBA is valuable. Small animal cohort and multiple arterial/section observations per animal limit precision and independence. Histological injury/emboli percentages are section-level findings.	Describes these devices in a porcine model; does not establish a class-wide clinical safety advantage or patient embolization rate.
[15] Clever et al., 2016	Porcine coronary coating/dose development study; crystalline and amorphous sirolimus coatings with uncoated controls	Acute tissue transfer, serial bulk tissue sirolimus, 1-month QCA and histomorphometry.	Direct formulation comparisons and chemical/histological endpoints support platform development. Healthy-animal biology, short follow-up and experimental dose/formulation variation limit clinical transfer.	Bulk tissue retention includes deposited material and does not quantify intracellular target engagement; evidence applies to studied formulations, not all SCBs.

Report	Design and sample	Measured endpoints	Study-specific appraisal	Inference
[16] Granada et al., 2016	Porcine coronary PK and histology development programme; Virtue porous balloon delivering nanoencapsulated sirolimus<br>130 pigs; 779 coronary segments: 753 PK segments and 26 biological-effect segments	Serial tissue/systemic sirolimus concentrations and 28-day histomorphometry in native, ISR and same-day stented sites.	Extensive PK sampling across time and tissues is a strength. Histological efficacy sample is far smaller than PK sample; multiple deliveries per animal and differing scenarios/doses complicate independence and comparisons. The report states protocols were not GLP-compliant.	Supports this porous delivery system's drug persistence and preclinical feasibility; cannot be generalized to surface-coated SCBs or treated as randomized human efficacy evidence.
[17] Kolachalama et al., 2013	Integrated ex vivo, porcine peripheral artery and computational study; zotarolimus balloon with proprietary excipients<br>18 pigs; four balloons per animal; separate bench experiments	Radiolabeled-drug partitioning/diffusion, whole-blood release, tissue zotarolimus uptake/clearance and model-predicted bound/free drug.	Parameters estimated experimentally and predictions compared with in vivo uptake strengthen mechanistic plausibility. Model and animal experiments lack plaque/calcification; multiple arterial treatments per pig and site differences require care. Binding fractions are modeled, not direct intracellular measurements.	Supports diffusion/binding mechanisms for this zotarolimus system; does not validate equivalent retention or clinical performance of sirolimus balloons.
[18] Verheye et al., 2017; SABRE	Prospective first-in-human single-arm multicentre ISR feasibility study; Virtue porous sirolimus balloon<br>50 patients at nine centres; 47 with 6-month angiography; 36 per protocol	30-day TLF, 6-month in-segment late lumen loss and 12-month clinical events.	Prospective multicentre follow-up supports feasibility. No concurrent comparator; small sample, incomplete angiographic follow-up and large per-protocol exclusion fraction limit causal efficacy claims. Favorable per-protocol results should not replace intention-to-treat outcomes.	Clinical feasibility supports the tested device only; LLL/events do not directly demonstrate tissue retention or superiority to other DCBs.
[19] Lemos et al., 2013	Preclinical technology-development report; phospholipid-encapsulated sirolimus in balloon and stent-plus-balloon formulations; rabbit and porcine models<br>Separate cohorts include four rabbits/eight arteries for labelled distribution, 14 pigs for dose finding, and eight rabbits for balloon-before-stent comparison	Drug release/tissue concentrations, fluorescent-labelled distribution, OCT and histological neointima/healing.	Dose/formulation controls and a randomized porcine dose-finding experiment strengthen development evidence. Multiple species and stenting sequences differ; fluorescence labels may alter particle properties. The rabbit balloon-before-stent comparison did not reduce already-low neointimal obstruction.	Demonstrates preclinical carrier feasibility, not clinical efficacy; labelled particle localization cannot by itself establish unmodified intracellular sirolimus exposure.

Report	Design and sample	Measured endpoints	Study-specific appraisal	Inference
[20] Costa et al., 2022	Phase I prospective multicentre single-arm first-in-human study; SELUTION SLR microsphere balloon; mixed native/restenotic lesions<br>56 patients/lesions; 45 cases assessed for angiographic binary restenosis at six months	Device/procedural success, 6-month QCA late lumen loss and clinical events.	Prospective multicentre enrollment provides early feasibility evidence. No randomized comparator, small cohort, mixed lesion types and incomplete angiographic follow-up limit precision and comparative interpretation.	Low observed event counts and LLL do not establish superiority, durable clinical safety, or the biological mechanism of microsphere retention.
[21] Heinrich et al., 2020	Comparative robotic abrasion bench experiment; eight commercial peripheral paclitaxel balloon types<br>24 DCBs; n=3 per type	Optical image/lightness changes representing coating-area loss during increasing friction in deflated/inflated states.	Standardized force application and identical test conditions facilitate comparative abrasion assessment. Room-temperature tests omit pulsatile fluid; optical changes can reflect structural changes/artifact, and measured coating-area loss is not chemically quantified drug mass.	Measures susceptibility to experimental abrasion, not embolic particle counts, clinical drug loss or tissue-transfer efficiency.
[22] Anbalakan et al., 2021	Integrated in vitro and computational sirolimus-DCB study with modeled atherosclerosis	Experimental release behavior and numerical predictions of uptake, retention and specific/nonspecific binding saturation.	Combination of experiments with comparison to existing in vivo data is a strength. Lesion effects and proposed exposure/release optimization are predictions conditional on transport/binding assumptions; independent prospective human validation was not assessed.	Supports mechanistic hypotheses; does not establish optimal clinical inflation duration, drug dose or lesion-specific efficacy.
[23] Anbalakan et al., 2022	In vitro/in silico comparison of urea and BTHC excipients with modeled lesion composition	Tracking loss, adhesion/release and drug uptake/distribution; modeled effects of noncalcified and calcified lesions.	Stage-specific comparison distinguishes tracking loss from subsequent uptake. Coating-specific conditions and lesion parameterization limit generalizability.	Experimental loss percentages and predicted calcification effects cannot rank marketed coronary DCBs or establish clinical lesion-preparation recommendations.
[24] Tscheuschner et al., 2025	Integrated porcine peripheral artery, bench tracking/sliding and computational study; PearlFlow paclitaxel/BTHC balloon<br>Four pigs: four ISR and four contralateral native SFA sites; eight in vivo balloons	LC–MS/MS plasma and target/upstream/downstream tissue paclitaxel; bench loss during insertion/bend passage and frictional tissue transfer.	Paired native/ISR sampling and separate pre-inflation plasma sampling strengthen localization of loss. One device, small sample, fixed treatment sequence, terminal assessment within one hour and an unusually long unprotected carotid-to-SFA route limit generalization.	Demonstrates acute drug redistribution in this model; cannot assign the same loss percentage to coronary procedures or infer clinical harm from upstream/drug concentrations.

Report	Design and sample	Measured endpoints	Study-specific appraisal	Inference
[25] Lee et al., 2024	Single-centre retrospective OCT cohort; SeQuent Please PCB in de novo coronary lesions.<br>328 patients/328 lesions; 31 TLF events; median follow-up 460 days.	Pre/post-PCI OCT calcium and dissection morphology; subsequent clinical TLF associations.	Serial OCT and blinded clinical outcome assessment; operator selection, exclusion of difficult OCT cases and bailout stenting, and few events create selection bias and possible model overfitting.	Calcium and medial-dissection findings are prognostic associations. Neither drug transfer nor a benefit from deliberately creating dissection was measured.
[26] Nijhoff et al., 2016	Post hoc comparison of sequential prospective, nonrandomized ISR cohorts; DIOR shellac-PCB versus IN.PACT Falcon urea-PCB.<br>45 patients: DIOR 20, IN.PACT Falcon 25.	Six-month angiographic late lumen loss, OCT neointimal volume and FFR.	Serial morphological and physiological measurements; small nonrandomized sequential groups, unblinded QCA/OCT, missing imaging and unmeasured confounding. Clinical events were not the principal efficacy measure.	Supports possible device-specific antirestenotic differences; cannot attribute a causal clinical benefit exclusively to excipient chemistry or infer tissue pharmacokinetics.
[27] Radke et al., 2011	Porcine coronary balloon angioplasty without added stents; four paclitaxel coatings (iopromide, ATEC, BTHC, lecithin) versus uncoated balloons. Commercial-platform equivalence not verified.<br>45 arteries in comparative healing experiment; separate BTHC PK measurements in 39 arteries.	28-day histology/histomorphometry and angiography; tissue paclitaxel concentrations for the BTHC formulation.	Multiple excipient comparators and direct BTHC drug measurement.	Healing responses differed but efficacy endpoints did not. Fibrin/inflammation are pharmacodynamic injury signals, not proof of beneficial clinical efficacy. Do not label the generic BTHC formulation as commercial Pantera Lux without verification.
[28] Yeh et al., 2024; AGENT IDE	Multicentre randomized superiority trial in coronary ISR; AGENT PCB versus uncoated balloon after successful preparation.<br>600 patients, 406 versus 194; 95.7% completed one-year follow-up.	One-year TLF (ischaemia-driven TLR, target-vessel MI or cardiac death).	Randomized, intention-to-treat analysis, blinded patients/core laboratory/events committee and independent oversight; operators unblinded, industry-funded, selective lesion criteria and uncoated rather than DES comparator.	Demonstrates clinical benefit of the tested complete platform versus uncoated balloon in eligible ISR. Does not establish superiority over DES or other DCBs, or isolate the excipient mechanism.

Report	Design and sample	Measured endpoints	Study-specific appraisal	Inference
[29] Xia et al., 2021	Bench simulated tracking/particle study and healthy porcine coronary study; Restore shellac-ammonium PCB versus SeQuent Please.<br>Bench tracking n=3 balloons/group; 6 swine/18 coronary treatments for 28-day histology; separate acute uptake assay reported 3 animals/6 vessel samples per group.	SEM morphology; pre-inflation wash-off; qualitative particles; 28-day injury score; tissue paclitaxel at 60 minutes.	Direct commercial-device comparison and analytical drug quantification; small groups, healthy vessels, qualitative particle assessment, blinding not reported, multiple arteries per animal and no demonstrated adjustment for clustering.	Lower bench wash-off does not prove superior clinical delivery or embolic safety. Sixty-minute tissue uptake was not significantly different; long-term retention and biologically available drug were not measured.
[30] Woolford et al., 2019	Laboratory process-development study of micropipette-coated paclitaxel/iopromide balloon prototypes.	Coating uniformity, thickness and texture by SEM; paclitaxel loading by HPLC; in vitro release.	Explicit manipulation of coating parameters with quantitative analytical assays.	Supports fabrication feasibility and sensitivity to coating conditions, not coronary tissue retention, in vivo safety or clinical equivalence to commercial products.
[31] Granada et al., 2014	Identical paclitaxel-iopromide balloon prototypes differing in crystalline/amorphous state; rabbit aortic PK, separate porcine coronary BMS-injury healing experiment.<br>44 rabbits for PK (4 vessels/time/group); 12 swine, 32 coronary arteries for healing.	LC-MS/MS of scraped endoluminal versus remaining vessel compartments through 28 days; separate coronary OCT/histology.	Matched formulation comparison, direct compartmental drug assay, independent analytical laboratory and blinded QCA/OCT. Small time-point samples; PK and healing measured in different species/beds; mechanical scraping is not microscopic localization.	Supports solid-state-dependent retention and healing in these models. Total extracted paclitaxel does not quantify free active drug, and surface scraping includes intima/endothelium. Cannot directly assign these PK values to commercial coronary balloons.
[32] Scheller et al., 2024	Four-centre randomized noninferiority trial; crystalline BHT-excipient SeQuent SCB versus SeQuent Please NEO PCB in de novo lesions.<br>70 patients, 35/group; follow-up angiography in 31 SCB and 28 PCB patients.	Six-month angiographic LLL; secondary clinical events through 12 months.	Blinded angiographic core laboratory; operators unblinded, clinical events assessed by local investigators, no independent events committee, incomplete angiographic follow-up, broad 0.35-mm noninferiority margin and unmodelled lesion-patient clustering.	Angiographic noninferiority is platform- and margin-specific. Small surrogate-endpoint trial cannot establish equivalent clinical safety, direct drug persistence or a class effect for sirolimus balloons.

Report	Design and sample	Measured endpoints	Study-specific appraisal	Inference
[33] Jiménez Díaz and Íñiguez Romo, 2023	Single-patient Clinical Picture; distal right coronary ACS lesion treated with a B. Braun crystalline sirolimus/BHT balloon. The report specifies manufacturer and formulation, without an exact commercial product name.<br>One 50-year-old woman; single 45-second inflation; repeat angiography and OCT at 28 days.	Qualitative serial OCT and angiography. Acute hyperechoic particles with shadowing; most disappeared by 28 days, with a few residual particles and maintained vessel patency.	Within-patient baseline, post-predilation, post-treatment and follow-up images document morphology. One uncontrolled case; no quantitative chemical tissue assay, histological confirmation, independent deposit quantification or comparative clinical design. Apparent absorption or endothelialization is an imaging interpretation.	Supports a temporal OCT description after this formulation. Appearance alone cannot identify chemical composition, bioavailable tissue concentration, release kinetics or a causal clinical benefit.
[34] Jiménez Díaz et al., 2026	Contextual human OCT publication describing crystal-like material after sirolimus-coated balloon angioplasty.	Cited to identify a published human OCT description.	Used as an illustrative imaging citation, not as independently appraised comparative or quantitative pharmacokinetic evidence.	No inference about a particular commercial platform, tissue concentration, drug activity, persistence interval or clinical efficacy is drawn from this citation.
[35] Scheller et al., 2022	Combined analysis of two parallel randomized trials; SeQuent SCB versus SeQuent Please PCB for DES-ISR.<br>101 patients in total.<br>Combined report of two parallel randomized trials.	Six-month angiographic LLL; secondary clinical events to 12 months.	Randomized direct comparison using identical parallel protocols; small pooled sample, surrogate primary endpoint and 0.35-mm noninferiority margin.	Supports angiographic performance of the tested SCB in DES-ISR; does not establish clinical equivalence or directly quantify drug-retention mechanisms.
[36] Liu et al., 2025	Prospective multicentre, assessor-blinded randomized coronary ISR trial; SeQuent SCB (sirolimus 4 micrograms/mm²) versus SeQuent Please NEO (paclitaxel 3 micrograms/mm²).<br>258 randomized patients with 285 lesions at 16 sites; 130 SCB and 128 PCB patients.	Nine-month angiographic in-segment late lumen loss; 12-month clinical outcomes. The reported angiographic result is not a tissue drug measurement.	Randomization, multicentre conduct and assessor blinding support the angiographic comparison. The endpoint is a surrogate with a 0.20-mm margin; the upper confidence bound (0.19 mm) lies close to that margin.	Supports angiographic noninferiority for these two named devices in ISR. It does not quantify human drug exposure, establish class-wide equivalence, or show equivalent clinical-event risk.

Report	Design and sample	Measured endpoints	Study-specific appraisal	Inference
[37] Chen et al., 2024	17-centre randomized noninferiority trial; central computer allocation after predilation; masked endpoint/core-laboratory assessment; BioAscend JWMS China biolimus 3 µg/mm² with polyethylene oxide vs SeQuent Please NEO paclitaxel 3 µg/mm² 280 randomized; 275 full analysis set patients; 230 patients (83.6%) with 9-month angiography	QCA in-segment LLL; OCT neointimal area in a substudy; clinical events. No human tissue PK.	Incomplete angiographic follow-up; small OCT subset; clinical endpoints underpowered; complex/high-risk lesions excluded. Angiographic noninferiority cannot establish clinical equivalence or a biolimus class effect.	Angiographic noninferiority applies to BioAscend versus SeQuent Please NEO in the studied ISR population. It is not clinical equivalence or a biolimus class effect.
[38] Byrne et al., 2025	Multicentre, assessor-blinded, 2:1 randomized noninferiority ISR trial; investigational Biosensors Biolimus A9-coated balloon versus SeQuent Please paclitaxel-coated balloon. 202 patients randomized at 20 centres in six countries: 135 biolimus balloon and 67 paclitaxel balloon.	Six-month quantitative angiographic in-segment percentage diameter stenosis; clinical target lesion failure through one year. The reported angiographic result is not a tissue drug measurement.	Randomized multicentre design with assessor blinding strengthens the angiographic comparison. The primary endpoint is a surrogate, the groups were unequal in size, and the clinical estimate is imprecise. The upper confidence bound exceeds the prespecified noninferiority margin.	The investigational biolimus device failed its angiographic noninferiority comparison with the named PCB. This result should not be transferred to other biolimus/limus devices or treated as proof of a particular release mechanism.
[39] Koch et al., 2024	Nonrandomized prospective Agent cohort compared with a historical randomized-trial control; AGENT PCB 2 µg/mm², citrate coating, versus first-generation SeQuent Please PCB 3 µg/mm², iopromide 262 patients/323 lesions: Agent 125/151 versus SeQuent 137/172; complete follow-up 84.0% versus 87.6%	Core-laboratory angiography and seven-year clinical TLR, death, MI and thrombosis.	Historical era differences; predilation 97.4% versus 80.8% and OCT 23.2% versus 0%; larger Agent vessels; clinical outcomes underpowered.	

Report	Design and sample	Measured endpoints	Study-specific appraisal	Inference
[40] Shin et al., 2025	Ten-centre open-label randomized angiographic noninferiority trial; Genoss shellac/vitamin-E PCB versus SeQuent Please NEO iopromide PCB; both 3 µg/mm² 204 randomized patients (102 per arm); six-month QCA available in 94 versus 89	Blinded-core-laboratory QCA LLL, late lumen enlargement and clinical events.	Selected successful preparation; open-label procedures; incomplete angiographic follow-up; primary per-protocol analysis; insufficient power for clinical safety equivalence.	The randomized result concerns these two coatings after successful preparation. It does not isolate an excipient mechanism or establish clinical equivalence.
[41] Tang et al., 2018; RESTORE SVD China	Multicentre randomized noninferiority trial; Restore PCB versus Resolute Integrity DES in 2.25–2.75-mm vessels; nested nonrandomized very-small-vessel cohort. 230 randomized patients, 116 DCB/114 DES; separate 32-patient registry.	Nine-month in-segment diameter stenosis (primary); one-year TLF.	Randomized multicentre comparison with prespecified stratification; powered for angiographic endpoint, few clinical events and restricted vessel size.	Angiographic noninferiority applies to the enrolled small-vessel population and complete device strategy. Does not establish superior coating transfer, clinical equivalence or general de novo-lesion efficacy.
[42] Ninomiya et al., 2023; TRANSFORM I	Prospective multicentre randomized noninferiority trial; MagicTouch SCB versus SeQuent Please Neo PCB; OCT-guided sizing in de novo small vessels. 121 patients/129 lesions; angiographic follow-up in 109 patients (90.1%) in per-protocol analysis. Parent trial of the OCT substudy in ref. 52.	Six-month angiographic net lumen gain; restenosis, lumen enlargement and angiography-derived physiology.	Direct randomized platform comparison and imaging-guided sizing; modest sample, angiographic primary endpoint and incomplete follow-up.	SCB failed prespecified noninferiority for net lumen gain. Results challenge assumed interchangeability of these devices, but do not isolate drug molecule from carrier/platform or directly test pharmacokinetics.

Report	Design and sample	Measured endpoints	Study-specific appraisal	Inference
[43] Gao et al., 2024; REC-CAGEFREE I	43-centre open-label randomized noninferiority trial; PCB strategy with rescue stenting versus planned thin-strut sirolimus-DES after successful preparation of noncomplex de novo lesions.<br>2,272 patients; 1,133 DCB/1,139 DES; rescue DES in 106 DCB patients (9.4%).	Intention-to-treat two-year device-oriented clinical composite.	Large clinical-endpoint trial; centralized web block randomization reported. Open-label treatment, single-country recruitment and selective lesion preparation limit transferability.	DCB strategy failed noninferiority and had more composite events. Does not isolate excipient performance or justify extrapolating selected small-vessel results to all de novo vessels.
[44] Latib et al., 2012; BELLO	Prospective multicentre randomized trial; paclitaxel DCB with provisional BMS versus paclitaxel-eluting stent in small vessels.<br>182 patients, 90 DCB/92 PES; bailout stenting in 20% of DCB-group lesions.	Six-month angiographic LLL (primary); restenosis, TLR and MACE.	Randomized direct strategy comparison; small sample, surrogate endpoint, baseline vessel-size imbalance, frequent bailout stenting and an older stent comparator.	Supports angiographic efficacy in the studied small-vessel setting. Cannot establish superiority over contemporary DES, clinical equivalence, or drug-delivery mechanism.
[45] Jeger et al., 2018; BASKET-SMALL 2	Multicentre open-label randomized noninferiority trial; DCB versus second-generation DES after successful preparation in vessels <3 mm.<br>758 randomized patients, 382 DCB/376 DES.<br>Parent trial; ref. 46 reports longer follow-up.	Twelve-month MACE; primary noninferiority comparison in per-protocol population.	Clinical primary endpoint and internet-based random allocation; open-label treatment, selected successful preparation and 4-percentage-point noninferiority margin.	Supports the tested strategy in eligible small native vessels, not unrestricted coronary use or device-class interchangeability; no direct drug-transfer measurement.
[46] Jeger et al., 2020; BASKET-SMALL 2 follow-up	Prespecified three-year follow-up of the same randomized BASKET-SMALL 2 cohort; DCB versus second-generation DES.<br>Same 758 randomized patients as ref. 45; not an independent trial.	Three-year MACE and individual clinical, thrombosis and bleeding outcomes; modified intention-to-treat analysis.	Prespecified longer follow-up preserves clinical relevance; original open-label/selective-entry limitations persist, and individual safety outcomes have wide confidence intervals.	Provides durability follow-up rather than independent replication. Similar event estimates do not demonstrate identical safety, explain drug retention or extend evidence beyond selected small vessels.

Report	Design and sample	Measured endpoints	Study-specific appraisal	Inference
[47] Mitsui et al., 2023	Single-centre retrospective nonrandomized OA cohort; Diamondback 360°OA with SeQuent Please PCB vs Xience/Synergy/Orsiro/Ultimaster DES<br>135 patients:43 DCB/92 DES; serial-OCT subgroup 14 event-free patients (7 per group)	OCT calcium/acute geometry and selected serial lumen-area response; 1-year clinical MACE. No tissue drug transfer measurements.	Strong treatment-selection confounding; groups differed in OA exposure; 14-person serial-OCT analysis excluded patients with primary events; sparse clinical events.	
[48] Oliveri et al., 2025; BENELUX-IVL analysis	Prospective multicentre observational IVL registry; post-IVL DCB versus DES chosen clinically.<br>579 patients: 45 DCB/534 DES; ISR in 60.0% versus 29.6%, respectively.	Procedural outcomes and clinical MACE through one year; available QCA/intravascular imaging.	Central imaging analysis and prospective registry; marked treatment-selection/lesion imbalance, small DCB cohort, unadjusted comparisons, incomplete imaging and no blinding.	Supports feasibility in selected balloon-crossable calcified lesions. Neither equivalence nor enhanced drug penetration after IVL was tested; no tissue drug assay.
[49] Gao et al., 2025 (DCB-BIF)	Multicentre randomized coronary strategy trial: DCB versus noncompliant balloon for a severely compromised side branch after main-vessel stenting; simple true bifurcations.<br>784 patients: 391 DCB, 393 noncompliant balloon; 22 centres.	One-year cardiac death, target-vessel MI or clinically driven TLR; procedural events; complete one-year follow-up reported.	Randomization and complete follow-up strengthen comparative outcome evidence. The composite difference was driven by MI; the authors themselves question the significance of frequent early periprocedural MI without revascularization. Selected anatomy and a main-vessel stent in both strategies restrict generalizability.	Supports the tested side-branch strategy; does not test DCB-only treatment of the whole bifurcation, coating transfer, mural concentration or platform-wide superiority.
[50] Scheller et al., 2012 (PACCOCATH-ISR I/II follow-up)	Long-term follow-up of randomized, double-blind multicentre trials: PACCOCATH paclitaxel balloon (3 micrograms/mm²) versus uncoated balloon for coronary ISR.<br>108 patients.<br>Long-term follow-up of PACCOCATH-ISR I/II.	Binary restenosis and clinical events over mean 5.4 years; reduction in events mainly reflected fewer repeat target-lesion interventions.	Randomized comparator, reported double-blinding and long follow-up support durability within this early platform. The small population cannot resolve uncommon harms; the uncoated comparator and historical ISR setting limit contemporary cross-device extrapolation. This is follow-up of the original trial cohorts, not independent additional patients.	Evidence for lasting clinical efficacy of the tested PACCOCATH lineage; no direct human tissue pharmacokinetics and no proof of identical efficacy across paclitaxel coatings.

Report	Design and sample	Measured endpoints	Study-specific appraisal	Inference
[51] Toelg et al., 2014 (DELUX)	Prospective international multicentre registry of Pantera Lux, a BTHC-excipient paclitaxel balloon; mixed BMS-ISR, DES-ISR and de novo lesions.<br>1,064 patients.	Device success and clinical MACE at 6 and 12 months, including lesion-category outcomes.	Large prospective real-world cohort describes performance in broader use. No randomized concurrent comparator; mixed lesion populations and operator selection prevent separation of device effect from case mix and procedural management.	Registry outcomes support feasibility and observed event rates; they do not isolate a BTHC-mediated transfer mechanism or demonstrate superiority over other coatings.
[52] Tobe et al., 2025 (TRANSFORM-I OCT substudy)	Single-centre imaging substudy of the randomized trial: MagicTouch SCB versus SeQuent Please NEO PCB in de novo small coronary vessels.<br>19 patients, 21 lesions: SCB 9 patients/11 lesions; PCB 10 patients/10 lesions.<br>Subset of ref. 42; not an independent trial.	Serial OCT lumen and dissection volumes immediately after PCI and at 6 months; between-group late lumen-volume loss p=0.06; dissection-volume interaction p=0.07.	Within-lesion serial imaging directly characterizes geometry. The very small, single-centre imaging subset has limited precision and may not represent the parent population. Significant change within one arm and nonsignificant change within the other is not evidence of a significant between-arm difference. Multiple lesions occur in some patients.	Exploratory platform-specific imaging differences, not established superiority, a drug-family effect or measured drug penetration/retention.
[53] Spaulding et al., 2026 (SELUTION DeNovo)	Open-label multicentre randomized noninferiority strategy trial: SELUTION SLR sirolimus-eluting balloon with provisional DES versus systematic DES; randomization before preparation.<br>3,341 randomized; full analysis set 3,323 (1,661 vs 1,662); per-protocol 3,194; 62 sites; 98.1% one-year follow-up in the full analysis set.	One-year TVF and clinical components; 20.7% of SEB-strategy participants received bailout DES. Primary ITT-principle analysis met noninferiority, whereas the PP sensitivity analysis did not.	Web-based concealed allocation, prespecified analysis and independent event adjudication are strengths. Open-label management, exclusions from the full analysis set, a 2.44% event-rate-dependent noninferiority margin, lack of systematic post-PCI biomarkers and nonstandardized antithrombotic regimens limit interpretation. One-year secondary-endpoint differences are exploratory and unadjusted for multiplicity.	Evidence concerns the SEB-plus-provisional-stent strategy, not direct SEB-versus-DES equivalence or proof of sustained human mural drug exposure. The PP result tempers the primary noninferiority conclusion; five-year assessment remains pending.
[54] Lee et al., 2024	Experimental everolimus-loaded gelatin-hydroxyphenyl propionic acid microgel balloon; in vitro release/cell assays, rabbit femoral drug transfer and porcine ISR model.	Drug loading and 30-day in vitro release; smooth-muscle/endothelial-cell responses; in vivo drug transfer/loss; porcine patency at 4 weeks.	Complementary formulation, cell and animal endpoints connect delivery engineering with biological response. Rabbit femoral delivery and short-term porcine ISR patency measure different stages in different models.	Supports preclinical microgel-delivery feasibility; 30-day release in vitro and four-week animal patency are not evidence of prolonged human coronary efficacy or tissue retention.

Report	Design and sample	Measured endpoints	Study-specific appraisal	Inference
[55] Kawai et al., 2024	SirPlux Duo nanoparticles containing paclitaxel:sirolimus 1:9; human VSMC assays, healthy pig femoral pharmacokinetics, injured rabbit iliac response and pig coronary/downstream histology; Prevail or AGENT comparators depending on experiment.<br>3 pigs for femoral pharmacokinetics; 9 rabbits (5 at day 5, 4 at day 28); 4 pigs for coronary/downstream assessment.	Mass-spectrometry drug concentrations at days 1/28; cell-cycle/BrdU assays; arterial histology; downstream myocardial injury and foreign material at day 28.	Direct concentration and histology measurements across complementary models are strengths. Small animal numbers, different comparator devices and overstretch conditions, no in vivo matched-dose single-drug nanoparticle arms and follow-up no longer than 28 days limit attribution. Downstream comparisons used multiple sections from four animals, limiting independent precision.	Findings support a specific experimental combination/coating; they neither isolate pharmacologic synergy from carrier/dose effects in vivo nor establish human safety superiority or clinical harm from comparator emboli.
[56] Cutlip et al., 2026	Randomized coronary ISR trial; SELUTION SLR sirolimus-eluting balloon versus operator-preselected usual care (repeat DES or balloon angioplasty).<br>418 randomized (210 SEB; 208 control); primary per-protocol population 390 (197 SEB; 193 control: 154 DES and 39 plain balloon angioplasty).	Clinical target lesion failure (cardiac death, target-vessel MI, or clinically driven target lesion revascularization) at one year. The reported clinical endpoint is not a tissue drug measurement.	Random allocation and a clinical primary endpoint strengthen interpretation. The primary question compared SEB with mixed usual care, used a 10-percentage-point noninferiority margin, and analyzed a per-protocol population. The prespecified single-layer DES comparison failed noninferiority.	Supports the reported platform-specific noninferiority result against mixed usual care. It does not establish equivalence to repeat DES, a class-wide sirolimus effect, or a coating-to-outcome mechanism.
[57] Yamamoto T. et al., 2020	Consecutive observational coronary DCB cohort with serial IVUS; de novo lesions.<br>46 patients, 54 lesions.	IVUS external elastic membrane, lumen and plaque volumes immediately after treatment and at 9 months; dissection index associated with later lumen-volume change.	Serial volumetric IVUS distinguishes vessel enlargement from plaque-volume change. Small selected cohort, no randomized non-DCB comparison and association-based regression restrict causal attribution.	LLE and its association with non-flow-limiting dissection are observed phenotypes. Greater penetration, adventitial drug exposure and a benefit from deliberately creating deep dissection were not measured.

Report	Design and sample	Measured endpoints	Study-specific appraisal	Inference
[58] Yamamoto M. et al., 2024	Retrospective single-centre SeQuent Please coronary cohort with serial OFDI after successful stent-free treatment of de novo lesions.<br>83 patients/108 lesions with follow-up, drawn from 113 patients/142 lesions with successful DCB treatment.	Lumen-volume change, plaque phenotype and dissection; LLE predefined as at least 10% volume increase; median follow-up 6.1 months.	Two readers blinded to QCA, serial measurements and reproducibility assessment are strengths. Operator selection, incomplete imaging follow-up, no independent core laboratory or uncoated-balloon arm, small plaque subgroups and limited OFDI penetration weaken causal/mechanistic inference. The study could not distinguish vessel enlargement from plaque regression in areas where the outer vessel boundary was not measurable.	Layered plaque and extensive medial dissection are associated predictors. The study did not quantify mural drug exposure, prove a remodeling mechanism or validate deliberate medial dissection as a treatment target.
[59] Kleber et al., 2015	Retrospective coronary de novo DCB cohort with a prespecified QCA protocol; reference segments used as an intra-patient control.	Postprocedure and follow-up minimum lumen diameter at mean 4.1 months, compared with reference-vessel changes.	Prespecified quantitative serial imaging and a within-patient reference comparison reduce some measurement variability. Selected successfully treated lesions, retrospective design and absence of a randomized uncoated-balloon arm prevent isolation of paclitaxel from mechanical injury, lesion selection and other remodeling influences.	Documents late lumen increase after treatment; QCA cannot identify tissue drug concentrations or prove which biological process caused that increase.
[60] Sogabe et al., 2021	Retrospective coronary cohort with serial OCT and angiography after paclitaxel-balloon treatment of de novo lesions without stenting.<br>91 patients, 95 lesions; mean follow-up 8.2 months.	LLE, vessel enlargement, dissection-flap healing and plaque/dissection-flap regression; lesion features associated with LLE.	Serial OCT permits direct morphological comparison. Retrospective selection of patients with follow-up imaging, small sample, absence of a randomized control and multiple lesions in some patients constrain causal interpretation.	Observed morphology and predictors do not measure drug penetration, establish a paclitaxel-specific causal pathway or justify intentional deep dissection.
[61] Gitto et al., 2026 (BAILOUT)	International 17-centre registry of consecutive patients receiving bailout DES after DCB angioplasty (2011–2024); historical DES performance-goal comparison.<br>733 bailout recipients, representing 5.5% of DCB-based PCI in contributing data.	One-year TLF, its components and stent thrombosis; TLF 7.0% (95% CI 5.2%–9.3%) compared with a historical 7.0% DES goal.	Multicentre data directly describe an important rescue scenario. The selected bailout population, long enrollment era and historical rather than contemporary randomized comparator allow confounding and case-mix differences.	Describes observed bailout outcomes; cannot establish equal safety to intended DES, a causal comparison of balloon drugs or a strategy of deliberately creating a dissection.

Report	Design and sample	Measured endpoints	Study-specific appraisal	Inference
[62] Asano et al., 2026	Prospective multicentre, open-label randomized exploratory pilot trial; Wolverine cutting balloon versus conventional semi-compliant/non-compliant balloon preparation. AGENT PCB used when the DCB-only strategy was selected.<br>210 patients randomized at 19 Japanese centres; 201 in the reported ITT population (101 cutting balloon, 100 standard balloon), after eight pre-PCI FFR-related exclusions and one consent withdrawal. Per-protocol population 191.	Composite immediate lesion-preparation endpoint before DCB deployment; independent-core-lab QCA and IVUS, operator-measured FFR, procedural outcomes and 30-day events. No drug-transfer assay, tissue concentration, or long-term remodeling result.	Central web randomization with minimization; QCA/IVUS core labs and event committee were blinded. Operators and patients were unblinded. FFR both guided treatment and formed part of the endpoint, creating acknowledged procedural/incorporation bias. The pilot was underpowered, nine randomized participants were excluded before PCI, and bailout decisions were discretionary. Selected native lesions (2.5–4.0 mm; length ≤28 mm) excluded several complex anatomies. The report covers acute and 30-day outcomes and one CB/DCB combination.	Shows exploratory differences in acute mechanical preparation for Wolverine/AGENT, with a neutral primary endpoint. It does not demonstrate better drug uptake, justify intentional medial dissection, establish superiority for clinical outcomes, or generalize to all preparation balloons/DCBs.
[63] Sagris et al., 2026	Systematic review/meta-analysis of randomized limus-versus-paclitaxel balloon comparisons in coronary ISR; sirolimus and biolimus platforms pooled.<br>Reported six RCTs; 1,208 patients (639 limus, 569 paclitaxel); mean follow-up 12 months.	Random-effects pooled clinical and angiographic outcomes, including TLR, TLF, restenosis and late lumen loss.	Registered protocol, independent duplicate screening, stated trial-level appraisal and ISR-focused eligibility are strengths. Sparse events, inclusion of a conference presentation, pooled limus drugs/coatings, inconsistent imaging and incompletely described preparation restrict platform attribution.	Nonsignificant pooled differences do not demonstrate equivalence or noninferiority. Low statistical heterogeneity does not remove biological/platform heterogeneity; no tissue transfer or retention is measured by this synthesis.
[64] Shin et al., 2024	Systematic review/meta-analysis combining randomized and observational PCB-versus-SCB comparisons in coronary ISR and de novo lesions.<br>Five randomized trials and three observational studies; 1,861 patients (889 PCB, 972 SCB).	Random-effects TLF at 9–12 months and angiographic late loss, diameter stenosis and minimum lumen diameter at 6–9 months.	Broad comparative synthesis includes clinical and angiographic endpoints. Mixed study designs and ISR/de novo populations introduce confounding and clinical heterogeneity.	Pooled nonsignificant TLF differences are not proof of device or drug-family equivalence. Angiographic results cannot identify the coating mechanism responsible or justify transfer of DES drug rankings to DCBs.

Report	Design and sample	Measured endpoints	Study-specific appraisal	Inference
[65] Sedhom et al., 2024	Random-effects meta-analysis of randomized limus-versus-paclitaxel balloon comparisons; coronary ISR and de novo lesions pooled.<br>Six RCTs; 821 patients (446 limus, 375 paclitaxel); mean clinical follow-up 13.4 months.	Clinically driven TLR and other clinical endpoints; angiographic binary restenosis, late loss, minimum lumen diameter and LLE.	RCT-only pooling strengthens comparative design. Small underlying trials, limited clinical-event power, mixed ISR/de novo lesions and multiple limus platforms limit certainty. TLR RR 1.32 (95% CI 0.84–2.08) is compatible with clinically meaningful differences.	Late angiographic differences may describe these included platforms/populations; they do not establish a universal drug-family mechanism or clinical equivalence.
[66] Haase et al., 2022	Series of standardized preclinical experiments conducted 2007–2022; pooled control and paclitaxel groups; Experimental balloons coated with arsenic trioxide, betamethasone dipropionate, bortezomib, EGCG, fantolon, methotrexate or thalidomide; paclitaxel controls QCA arteries: arsenic 8, betamethasone 12, bortezomib 10, EGCG 5, fantolon 10, methotrexate 38, thalidomide 10, uncoated 81, paclitaxel 48.	Analytical balloon/tissue drug content; blinded QCA at four weeks; histomorphometry, injury and inflammation scores	Different experiments, formulations and doses pooled; several drug-transfer values not reported; histology incomplete for some drugs; multiple arteries per pig without stated animal-level clustering adjustment; four-week juvenile nonatherosclerotic model. Acute transfer is insufficient evidence of antiproliferative efficacy.	Acute arterial drug recovery is not an efficacy endpoint. Pooled animal experiments cannot isolate the contribution of drug, coating, dose or model, or establish human clinical benefit.
[67] Renon et al., 2025	Narrative methodological review of DCB computational models and clinical translation; contextual secondary evidence.<br>Not applicable: no new experimental or clinical cohort.	Reviews existing transport/mechanical models and their assumptions; no new human pharmacokinetic measurements or prospective prediction-validation study.	Explicit comparison of dimensionality, geometry, balloon representation, parameter sources and validation gaps aids contextual interpretation. The models reviewed commonly simplify tissue/balloon geometry and use parameters from different vascular beds or nonhuman experiments; integrated patient-specific deployment, transport and remodeling validation remains lacking.	Useful to define testable hypotheses and modeling limitations, not independent evidence validating any named platform, human tissue exposure or prospective clinical decision support.

Abbreviations: BMS, bare-metal stent; BTHC, butyryl-tri-hexyl citrate; DCB, drug-coated balloon; DES, drug-eluting stent; EPC, endothelial progenitor cell; FFR, fractional flow reserve; hCASMC, human coronary artery smooth-muscle cell; ISR, in-stent restenosis; ITT, intention-to-treat; IVL, intravascular lithotripsy; IVUS, intravascular ultrasound; LC-MS/MS, liquid chromatography with tandem mass spectrometry; LLE, late lumen enlargement; LLL, late lumen loss; MACE, major adverse cardiac events; MI, myocardial infarction; OA, orbital atherectomy; OCT, optical coherence tomography; OFDI, optical frequency-domain imaging; PCB, paclitaxel-coated balloon; PCI, percutaneous coronary intervention; PK, pharmacokinetics; PP, per-protocol; QCA, quantitative coronary angiography; RCT, randomized controlled trial; SCB, sirolimus-coated balloon; SEB, sirolimus-eluting balloon; SEM, scanning electron microscopy; SFA, superficial femoral artery; TLF, target-lesion failure; TLR, target-lesion revascularization; TVF, target-vessel failure.

Supplementary Methods S1

Search and evidence assessment during revision

This supplement documents the additional literature search and evidence assessment performed on 9 September 2026 during revision of the coronary DCB narrative review. It supplies exact queries, counts and record dispositions. The original May searche remain separate from this newly documented process.

1 Scope and source selection

The search addressed coronary DCB engineering, drug transfer or retention, tissue pharmacokinetics, quantitative angiographic or intravascular imaging response and named-platform clinical comparisons. Original experimental reports, quantitative imaging cohorts and clinical comparisons were candidates for report retrieval. Reviews, consensus statements, protocols and descriptive cases were recorded as contextual sources. Peripheral clinical-outcome studies without relevant drug-delivery experiments, non-DCB treatment reports and non-English publications were excluded. Conference abstracts, preprints and unpublished reports were not eligible.

2 Search execution and exact final queries

Database and interface: PubMed was searched using the NCBI E-utilities API, with ESearch used to identify records and EFetch to retrieve bibliographic data and available abstracts. Execution date: 9 September 2026. Publication filter: 1 January 1900 through 9 September 2026.

Publication-date filtering can include an earlier electronic publication with a later print date and does not recreate the database contents on a historical date.

Query A Engineering pharmacokinetics and imaging

```
(coronary[Title/Abstract] OR coronaries[Title/Abstract]) AND ("drug-coated balloon*"[Title/Abstract] OR "drug-eluting
balloon*"[Title/Abstract] OR "paclitaxel-coated balloon*"[Title/Abstract] OR "sirolimus-coated balloon*"[Title/Abstract] OR
"sirolimus-eluting balloon*"[Title/Abstract] OR "paclitaxel-eluting balloon*"[Title/Abstract]) AND (coating*[Title/Abstract] OR
excipient*[Title/Abstract] OR pharmacokinetic*[Title/Abstract] OR "drug transfer"[Title/Abstract] OR "drug
retention"[Title/Abstract] OR "tissue retention"[Title/Abstract] OR "tissue uptake"[Title/Abstract] OR
engineering[Title/Abstract] OR "drug delivery"[Title/Abstract] OR "optical coherence tomography"[Title/Abstract] OR
"intravascular ultrasound"[Title/Abstract] OR OCT[Title/Abstract] OR IVUS[Title/Abstract] OR carrier*[Title/Abstract] OR
nanocarrier*[Title/Abstract] OR reservoir*[Title/Abstract] OR microreservoir*[Title/Abstract] OR crystall*[Title/Abstract] OR
encapsulat*[Title/Abstract] OR biodegrad*[Title/Abstract] OR release[Title/Abstract]) AND ("1900/01/01"[Date - Publication] :
"2026/09/09"[Date - Publication])
```

Query B Named coronary balloon platforms

```
(coronary[Title/Abstract] OR coronaries[Title/Abstract]) AND ("drug-coated balloon*"[Title/Abstract] OR "drug-eluting balloon*"[Title/Abstract] OR "paclitaxel-coated balloon*"[Title/Abstract] OR "sirolimus-coated balloon*"[Title/Abstract] OR "sirolimus-eluting balloon*"[Title/Abstract] OR "paclitaxel-eluting balloon*"[Title/Abstract]) AND (SeQuent[Title/Abstract] OR AGENT[Title/Abstract] OR MagicTouch[Title/Abstract] OR SELUTION[Title/Abstract] OR Pantera[Title/Abstract] OR RESTORE[Title/Abstract] OR DIOR[Title/Abstract] OR IN.PACT[Title/Abstract] OR Danubio[Title/Abstract] OR Elutax[Title/Abstract] OR "SeQuent SCB"[Title/Abstract]) AND ("1900/01/01"[Date - Publication] : "2026/09/09"[Date - Publication])
```

3 Retrieval and screening results

The 566 records included 36 without abstracts. All 566 received a title/abstract disposition. The 282 retrieval candidates received a documented source disposition. The 85 complete-report assessments comprise 76 primary-relevant, 7 contextual and 2 excluded reports. The 197 not-retrieved reports remain distinguishable from fully appraised studies. These counts describe reports, not independent patient cohorts or an exhaustive set of studies included in a systematic review.

Stage	Records	Interpretation
Query A	442	Native PubMed count
Query B	199	Native PubMed count
Overlap	75	Identical PMIDs across the two queries
Unique records screened	566	All retrieved; 542 distinct nonempty DOIs
Contextual at title/abstract stage	233	Reviews, protocols and descriptive reports
Excluded at title/abstract stage	51	Scope or language reasons recorded individually
Candidates for complete report retrieval	282	268 potential primary reports plus 14 insufficiently described records
Complete reports assessed	85	77 newly accessed plus 8 previous full-text appraisals reused
Complete reports not retrieved	197	No completed full-method appraisal
Primary evidence after full-text assessment	76	Recorded in the evidence map
Context after full-text assessment	7	Retained as context

4 Role of the search in the narrative synthesis

Seven reports were added to the main bibliography because they directly qualified a statement already within the review's scope: SIBLINT-ISR for the crystalline sirolimus platform; BIO ASCEND ISR and REFORM for distinct biolimus devices; Genoss for a randomized paclitaxel-coating comparison; NATURE for lesion-preparation evidence; SELUTION4ISR for comparator-specific clinical noninferiority; and Haase et al. for the distinction between acute drug recovery and efficacy. All 67 references are numbered in order of first appearance in the revised manuscript, including its tables.
